# Working memory network dysfunction in bipolar I vs. bipolar II disorder: a systematic review of task-fMRI evidence

**DOI:** 10.3389/fpsyt.2026.1800042

**Published:** 2026-06-16

**Authors:** Hao Yu, Tingting Wang, Yi Wang, Junfan Liang, Qing Zou, Jiaqi Xiang, Yang Lu, Kezhi Liu

**Affiliations:** 1 Department of Psychiatry, Affiliated Hospital of Southwest Medical University, Luzhou, Sichuan, China; 2 Department of Psychosomatic Medicine, The First Affiliated Hospital of Nanchang University, Nanchang, Jiangxi, China

**Keywords:** bipolar I disorder, bipolar II disorder, systematic review, task-fMRI, working memory

## Abstract

**Background:**

Working memory impairment is one of the core cognitive phenotypes in bipolar disorder. However, whether the task-based functional magnetic resonance imaging mechanisms underlying working memory processing are consistent between bipolar disorder type I (BD-I) and type II (BD-II) remains unclear. This systematic review summarizes task-based fMRI evidence, with a focus on characterizing the neural correlates of working memory in BD-II and comparing them with BD-I.

**Methods:**

Following the PRISMA 2020 guidelines, PubMed, Embase, and Web of Science were systematically searched for studies published between January 2011 and June 2025. Eligible studies included task-based fMRI investigations in adults using working memory paradigms that separately reported results for BD-I and BD-II. A total of 22 studies were included for qualitative synthesis.

**Results:**

Across 22 task-based fMRI studies, the qualitative evidence suggests potentially different patterns of working-memory network engagement in BD-I and BD-II; however, subtype-specific inference is constrained by the very limited BD-II literature, with only three studies including BD-II samples (one BD-II-only study and two direct BD-I vs BD-II comparisons). In BD-I, studies more often reported altered or less efficient recruitment of executive-control regions/networks and reduced task-related suppression of default-mode regions under higher cognitive load and/or emotional distraction. In BD-II (based on sparse evidence), euthymic samples were generally reported to show relatively preserved recruitment and performance, whereas depressive-state or higher-demand conditions were associated with attenuated load-related upregulation. In a single emotional-interference paradigm, BD-II showed stronger inverse DLPFC–amygdala coupling, which may reflect context-specific modulation of fronto-limbic interactions.

**Conclusion:**

The current task-fMRI literature provides preliminary, hypothesis-generating indications that BD-I and BD-II may not be fully captured by a simple severity-continuum account, but firm subtype-specific conclusions are not yet warranted given the scarcity of BD-II studies and the limited number of direct BD-I/BD-II comparisons. Across studies, BD-I findings have more often been interpreted within neural inefficiency/limited-scalability accounts and reduced task-related DMN suppression, whereas BD-II findings—based on sparse evidence—have been reported as more state- and context-dependent. Larger, harmonized studies with direct BD-I/BD-II comparisons and mood-state stratification are needed to test these provisional patterns.

## 1 Introduction

Bipolar disorder (BD) is a chronic and severe psychiatric condition associated with substantial functional disability and premature mortality, particularly due to elevated suicide risk (1, 2). It remains a leading contributor to global disability, markedly reducing quality of life and long-term socio-occupational attainment (3, 4). Although BD is defined by the occurrence of manic or hypomanic episodes, symptomatic remission does not necessarily translate into functional recovery. This dissociation is partly attributable to cognitive impairment, which is increasingly recognized as a core feature of BD and a stronger predictor of functional outcome than residual mood symptoms (5, 6).

Working memory (WM)—the temporary maintenance and manipulation of information supporting complex cognition (e.g., comprehension, learning, reasoning)—has long been conceptualized as a central cognitive construct (7). WM impairment is frequently reported in BD and may persist even during periods of emotional stability, supporting its role as a core cognitive phenotype of the illness (8, 9). This makes WM a strong target for a network-based synthesis of task-fMRI findings. WM paradigms (e.g., n-back; delayed match-to-sample, DMTS) permit parametric manipulation of cognitive load and can be combined with emotional distraction, offering a principled way to examine affect–cognition interactions relevant to BD (10–12). In addition, WM tasks elicit highly reproducible engagement of large-scale networks, which provides a common mechanistic framework for integrating results across studies and comparing BD subtypes (13). Over the past decades, functional magnetic resonance imaging (fMRI) has provided key insights into the neural substrates of these deficits, repeatedly implicating aberrant activation patterns within the central executive network (CEN) and the default mode network (DMN) (14, 15). Meta-analyses of WM in healthy individuals consistently delineate a robust and reproducible task-positive network. This network encompasses lateral prefrontal and posterior parietal cortices, and further extends to the anterior insula/dorsal anterior cingulate cortex (ACC), as well as the dorsal premotor and pre-supplementary motor areas. Collectively, these findings suggest that efficient WM processing depends on the coordinated recruitment and integration of multiple executive-control and attentional systems, rather than being subserved solely by the canonical dorsolateral prefrontal–posterior parietal circuit (10, 13, 16).

Neurobiologically, successful WM performance depends on coordinated interactions among large-scale intrinsic networks. During task engagement, the brain typically recruits the CEN—anchored in the dorsolateral prefrontal cortex (DLPFC) and posterior parietal cortex (PPC)—while suppressing the DMN, particularly midline hubs such as the medial prefrontal cortex (mPFC) and posterior cingulate cortex (PCC)/precuneus (17). This anti-correlated dynamic is critical, as insufficient DMN suppression may introduce neural interference and contribute to attentional lapses. The CEN–DMN antagonism is further regulated by the salience network (SN), anchored in the anterior insula and dorsal ACC, which has been proposed to detect behaviorally relevant events and facilitate switching between internally oriented DMN activity and externally oriented CEN engagement (18), a control architecture later formalized in the triple-network model linking SN–CEN–DMN interactions to psychopathology (19). Consistent with cognitive control accounts, the CEN’s conceptual origins trace back to Baddeley’s WM model (20) and align with prefrontal “top-down” control theory (21), while network imaging work delineates the CEN as a fronto-parietal lateral control circuit with extensions to frontopolar/vlFPC regions implicated in strategy integration, inferior parietal lobule (IPL) nodes supporting maintenance/buffering, and premotor areas involved in response preparation (19, 22–25). Beyond cortical networks, subcortical circuits including limbic and striatal systems may operate as an “affective–cognitive gate”, filtering distractor signals to protect limited cognitive resources (26). In parallel, the dorsal attention network (DAN) supports sustained top-down attentional orienting under increasing task demands, and cingulo-opercular systems are often linked to stable task-set maintenance across time (27, 28). The DMN tends to operate coherently during rest/internal mentation and typically requires suppression during externally oriented goal-directed tasks, with broader involvement of ventral/rostral anterior cingulate, lateral parietal cortices (adjacent to the angular gyrus), temporal poles, and medial temporal lobe structures (29–32). In addition to these large-scale systems, WM performance can draw on modality-specific support regions; for example, the superior temporal gyrus (STG) has been linked to phonological rehearsal processes (33). In Bipolar I Disorder (BD-I), converging evidence suggests disruptions in both executive recruitment and task-related DMN suppression (34, 35).

Importantly, the neurobiological profile of BD-I appears heterogeneous. While DLPFC hypoactivation is commonly reported, task-based studies have also described paradoxical hyperactivation in executive regions, potentially reflecting inefficient compensatory recruitment under specific cognitive loads, state effects, or illness-related moderators. This intra-subtype variability has been interpreted within a “neural inefficiency” framework, in which prefrontal recruitment may show a non-linear (inverted U-shaped) relationship with task demand—relatively greater activation at lower loads, followed by reduced activation when cognitive demands exceed available capacity (36–39).

Despite these advances, a major limitation in the literature is the persistent tendency to treat BD as a homogeneous entity, potentially obscuring subtype-specific mechanisms. Neuroimaging models have historically been derived predominantly from BD-I cohorts, whereas Bipolar II Disorder (BD-II) has been underrepresented or conflated with BD-I in mixed-sample designs (40, 41). This grouping approach often assumes BD-II is a milder form; however, clinical evidence indicates BD-II confers a substantial and distinct burden, characterized by more frequent and persistent depressive episodes, higher rates of mixed features and rapid cycling, and marked functional impairment, with suicide risk comparable to—or in some reports exceeding—that of BD-I (42–44). BD-II is also more prevalent among women and frequently co-occurs with anxiety disorders, personality pathology, and multiple somatic comorbidities, further underscoring its clinical distinctiveness (44). Consequently, it remains unclear whether WM deficits in BD-II reflect the same neural dysfunctions described in BD-I or instead follow a distinct pathophysiological trajectory linked to its predominant depressive polarity (45). Direct neuroimaging comparisons between BD-I and BD-II remain sparse and yield competing interpretations. Some evidence supports a severity-continuum account, suggesting BD-II shows an intermediate pattern of prefrontal dysfunction between healthy controls and BD-I (38). In contrast, other studies point to neural dissociation, with relatively preserved DLPFC activation but selective alterations in frontopolar and parietal regions in BD-II, implying a distinct mechanistic profile (46). Moreover, while reduced task-related DMN suppression is often highlighted as a hallmark in BD-I (47), it remains uncertain whether this biomarker generalizes to BD-II. Crucially, it is also unknown whether BD-II exhibits the same vulnerability to emotional interference as BD-I, or if it retains a distinct capacity for top-down regulation when facing affective distractors.

WM dysfunction is not unique to BD and has also been widely reported in major depressive disorder (MDD). Behavioural evidence from a systematic review and meta-analysis of the n-back task suggests that individuals with unipolar depression show reduced accuracy at higher memory loads (1–3-back) and prolonged response times across loads, consistent with load-sensitive executive WM impairment (48). Task-based neuroimaging meta-analyses further implicate altered recruitment of cognitive control circuitry (including anterior insula and rostral ACC) during cognitive–emotional challenges in MDD (49). Focusing specifically on WM tasks, a coordinate-based meta-analysis reported abnormal activation patterns in both task-positive control regions and default-mode regions in MDD, including disorder-specific reductions in middle frontal gyrus engagement (50). In addition, reduced suppression of the DMN during WM has been observed in remitted MDD and linked to rumination (51). Given the predominantly depressive polarity of BD-II, this MDD literature provides a clinically relevant comparator for interpreting whether WM-related findings in BD-II.

Given these inconsistencies, this systematic review synthesizes task-based fMRI studies that report BD-I and BD-II findings separately to describe WM-related neural patterns in BD-II and to assess whether the qualitative evidence more strongly aligns with subtype differentiation or a severity-continuum account. To concurrently capture the effects of both cognitive load and emotional context, we have organized the review according to experimental paradigms: standard n-back tasks and their emotionally salient variants (emotional n-back), are analyzed together, supplemented by examination of the DMTS paradigm where inferences about specific encoding and maintenance phases can be drawn. Across all paradigms, our focus extends beyond the classical balance between the CEN and the DMN to also encompass the broader control architecture responsible for coordinating neural recruitment, suppression, and gating—including networks such as the SN, DAN, and prefrontal-limbic coupling.

## 2 Methods

### 2.1 Systematic review framework

This systematic review was conducted in accordance with the Preferred Reporting Items for Systematic Reviews and Meta-Analyses (PRISMA) 2020 guidelines (52). The study protocol was designed to synthesize task-based fMRI evidence on WM and to compare WM-related neural mechanisms between BD-I and BD-II disorders. The review protocol was developed *a priori* but was not registered in an external registry (e.g., PROSPERO). We acknowledge that the absence of prospective registration may reduce transparency and increase the risk of selective reporting. To enhance reproducibility, complete database-specific search strategies, exact search dates, and database-level retrieval counts are provided in the **Supplementary Material** (**Supplementary Table 1**).

#### 2.1.1 Search strategy and data sources

To structure the search, we applied a PICO (53) framework adapted for cross-sectional neuroimaging studies, operationalizing the “Intervention” component as an “Indicator” (WM task paradigms), consistent with prior fMRI systematic reviews (54).

We searched PubMed, Embase, and Web of Science from January 1, 2011 to June 30, 2025, combining controlled vocabulary (e.g., MeSH/Emtree terms) with free-text Title/Abstract terms. We restricted eligibility to studies published from January 2011 onward because the primary aim of this review was to examine subtype-related (BD-I vs BD-II) differences in task-based fMRI correlates of WM. In earlier task-fMRI literature, bipolar samples were frequently reported as a single group without consistent subtype stratification or sufficient subtype-specific results to support our subtype-focused synthesis. Accordingly, the 2011 start date was selected *a priori* as a pragmatic boundary to prioritize studies with more complete diagnostic characterization and reporting relevant to the current review question. Search terms were organized into four concept domains: Participants (BD-I/BD-II), Indicator (WM paradigms such as n-back, Sternberg/DMTS, spatial/verbal WM), Comparator (healthy controls), and Outcomes (task-based fMRI/BOLD activation or connectivity). Searches were restricted to English-language studies in adults (≥18 years), and reference lists of relevant articles were manually screened. The complete database-specific search strategies (as executed), exact search dates (day/month/year), and database-level retrieval counts prior to deduplication are provided in the **Supplementary Material** (**Supplementary Table 1**). Searches were last run on 12/10/2025 (12 October 2025) and yielded 146 records from PubMed, 126 from Embase, and 194 from Web of Science (prior to deduplication).

#### 2.1.2 Subtype-specific eligibility criteria

Records retrieved from all databases were exported and de-duplicated prior to screening. Two reviewers independently screened titles and abstracts, followed by full-text assessment against eligibility criteria. The review process was designed to be fully consensus-based: any disagreements were resolved through discussion between the two reviewers. In the few instances where consensus could not be reached, a third senior reviewer was consulted for a final decision. Given this consensus-oriented workflow, a formal inter-rater reliability statistic (e.g., Cohen’s κ) was not calculated; however, the low number of disputes requiring third-party adjudication serves as a qualitative indicator of high initial agreement between the primary reviewers.The selection process is summarized using a PRISMA 2020 flow diagram.

Studies were included if they: (a) recruited adult participants (≥18 years) with a confirmed diagnosis of BD-I and/or BD-II according to DSM or ICD criteria; (b) employed task-based fMRI to measure BOLD signal changes during WM performance; and (c) reported neuroimaging results for BD-I and BD-II separately, or provided a direct head-to-head comparison. Studies that included mixed BD samples were eligible only if subtype-specific neuroimaging outcomes could be extracted or were reported explicitly.

Exclusion criteria were: (a) non-original research (reviews, meta-analyses, editorials, case reports); (b) studies in which BD subtypes were conflated, unspecified, or not separable; (c) pediatric samples or samples with major neurological comorbidity (or history of severe head trauma); and (d) paradigms primarily indexing response inhibition or sustained attention (e.g., Stroop, Go/No-Go) rather than WM operations, to maintain focus on executive components of WM. Emotion-interference WM variants (e.g., emotional face distractors during n-back) were eligible and were synthesized alongside standard n-back tasks to reflect affect–cognition interaction under WM load.

#### 2.1.3 Formal search string

The final optimized search string was as follows:

(“Bipolar Disorder”[MeSH] OR “bipolar i”[tiab] OR “bipolar ii”[tiab] OR “bipolar 1”[tiab] OR “bipolar 2”[tiab] OR “bd-i”[tiab] OR “bd-ii”[tiab] OR “bipolar type i”[tiab] OR “bipolar type ii”[tiab] OR “bipolar I disorder”[tiab] OR “bipolar II disorder”[tiab] OR “manic depression”[tiab] OR “manic-depressive illness”[tiab]) AND (“Memory, Short-Term”[MeSH] OR “Working Memory”[tiab] OR “WM”[tiab] OR “n-back”[tiab] OR “n back”[tiab] OR “nback”[tiab] OR “Sternberg”[tiab] OR “delayed matching to sample”[tiab] OR “DMTS”[tiab] OR “delayed response task”[tiab] OR “spatial working memory”[tiab] OR “verbal working memory”[tiab]) AND (“Functional Neuroimaging”[MeSH] OR “Magnetic Resonance Imaging”[MeSH] OR “fMRI”[tiab] OR “functional MRI”[tiab] OR “task-based fMRI”[tiab] OR “task fMRI”[tiab] OR “functional magnetic resonance”[tiab] OR “BOLD”[tiab] OR “brain activation”[tiab] OR “neural activation”[tiab]).

### 2.2 Data extraction and quality assessment

Data from eligible studies were independently extracted by two researchers using a standardized form. Extracted variables included study characteristics (author, year), sample demographics (sample size, age, gender, mood state), task parameters (paradigm, load), and neuroimaging findings (anatomical regions and direction of activation/connectivity changes). Discrepancies were resolved by discussion and when necessary, consultation with a third reviewer. All extracted data were cross-checked for completeness and accuracy prior to synthesis.

Study quality was evaluated using a dual-criteria framework capturing both general methodological rigor and fMRI-specific technical validity. General study quality was assessed with the Newcastle–Ottawa Scale (NOS) (55), applying the case–control NOS to cross-sectional case–control designs and the cohort NOS to the longitudinal cohort study. fMRI-specific methodological rigor was assessed using a checklist adapted from established fMRI reporting recommendations (56) and neuroimaging best practice considerations (57) (**Table 1**, **Supplementary Table 3**). The checklist covered five domains: (1) multiple-comparisons correction, (2) head-motion control, (3) sample size and eligibility reporting (transparency), (4) in-scanner task performance reporting, and (5) task engagement/manipulation-check evidence (neural and/or behavioral). Each item was scored binarily (0/1; total 0–5), with “not reported/unclear” scored as 0. Studies were rated High Quality if they met both thresholds (NOS ≥ 7 stars and fMRI technical score ≥ 4), Moderate Quality if they met only one threshold, and Low Quality if they met neither. In addition, studies reporting whole-brain results exclusively at uncorrected thresholds without any multiple-comparisons correction and without a clearly justified corrected ROI/SVC alternative were classified as Low Quality regardless of NOS score (57). Corrected whole-brain inference included voxel-wise FWE/FDR, permutation-based correction, TFCE, or clearly reported cluster-level correction; corrected ROI/SVC approaches were considered acceptable when ROIs were a priori/independently defined and the correction procedure was clearly described.

**Table 1:** fMRI-specific methodological checklist.

Category	Item	Criteria for 1 point	Criteria for 0 points	Rationale
Statistical Analysis	Multiple Comparisons Correction	Whole-brain inference is corrected (voxel-wise FWE/FDR; permutation/TFCE; cluster-level with reported cluster-forming threshold + corrected cluster significance). ROI/SVC acceptable only with a priori/independent ROI and clearly described correction.	Whole-brain uncorrected thresholds OR method not stated/unclear.	Controls false positives; improves interpretability and reproducibility.
Data Quality	Head Motion Control	Motion handled with reportable criteria/QC (exclusion thresholds and exclusions; nuisance regressors + QC summary; scrubbing/advanced denoising with criteria).	No motion QC/procedures, or vague “motion corrected” only.	Motion confounds activation/connectivity; group/state differences can bias clinical findings.
Study Design	Sample Size and Eligibility Reporting (Transparency)	The final analyzed sample size (N) is clearly reported by group and/or timepoint/condition for the primary fMRI analyses and the study provides clear eligibility/inclusion–exclusion criteria for participant selection. If the manuscript explicitly indicates that participants were excluded after acquisition (e.g., due to motion, technical problems, or task non-compliance), the number excluded and reasons (or an explicit statement that no participants were excluded after acquisition) must be reported.	The final analyzed N is unclear for the primary fMRI analyses and/or eligibility/inclusion–exclusion criteria are insufficiently described; or Post-acquisition exclusions are explicitly mentioned but the number excluded and reasons (or a clear statement of no exclusions) are not reported.	Small samples lower power/precision and reduce replicability.
Behavioral Data	Task Performance Verification	Reports in-scanner accuracy/RT with sufficient detail; between-group/state comparability tested or controlled.	Behavioral data missing/insufficient; comparability unclear.	Without performance data, neural differences may reflect engagement/effort differences.
Validity	Task Engagement/Manipulation Check Evidence	Reports the primary within-task manipulation (e.g., high-load > low-load, 2-back > 0-back, parametric load) with sufficient detail (figure/table/peak coordinates or explicit text) in controls and/or the full sample; OR; If only group-difference results are presented, the study provides a clear manipulation check showing that participants performed the task as expected (e.g., appropriate behavioral summaries across load) and explicitly defines the task contrast(s) used for inference.	No primary task manipulation effect is shown/describable and no clear manipulation-check evidence is provided (cannot judge whether WM was effectively engaged)	Supports construct validity that the paradigm engaged WM circuitry.

FDR, False Discovery Rate; fMRI, Functional Magnetic Resonance Imaging; FWE, Family-Wise Error; N, Sample Size (Number of participants); QC, Quality Control; ROI, Region of Interest; RT, Reaction Time; SVC, Small Volume Correction; TFCE, Threshold-Free Cluster Enhancement; WM, Working Memory.

### 2.3 Data synthesis

Given the limited number of direct BD-I vs BD-II comparisons and substantial heterogeneity in paradigms, contrasts, and analytic approaches (e.g., varying n-back loads, ROI vs whole-brain strategies, and connectivity methods), a qualitative synthesis was conducted rather than a quantitative meta-analysis. Evidence was first organized by paradigm, grouping standard n-back studies together with emotion-interference variants (EFNBACK), and synthesizing DMTS studies separately to enable phase-specific inference (encoding vs maintenance) where available. Within each paradigm, findings were mapped onto large-scale network frameworks, focusing primarily on CEN recruitment and task-related DMN suppression while also extracting results relevant to salience/attention systems and fronto-limbic or subcortical modulatory circuits when reported, to support subtype-level mechanistic comparison.

## 3 Results

### 3.1 Literature search and selection process

**Figure 1:**
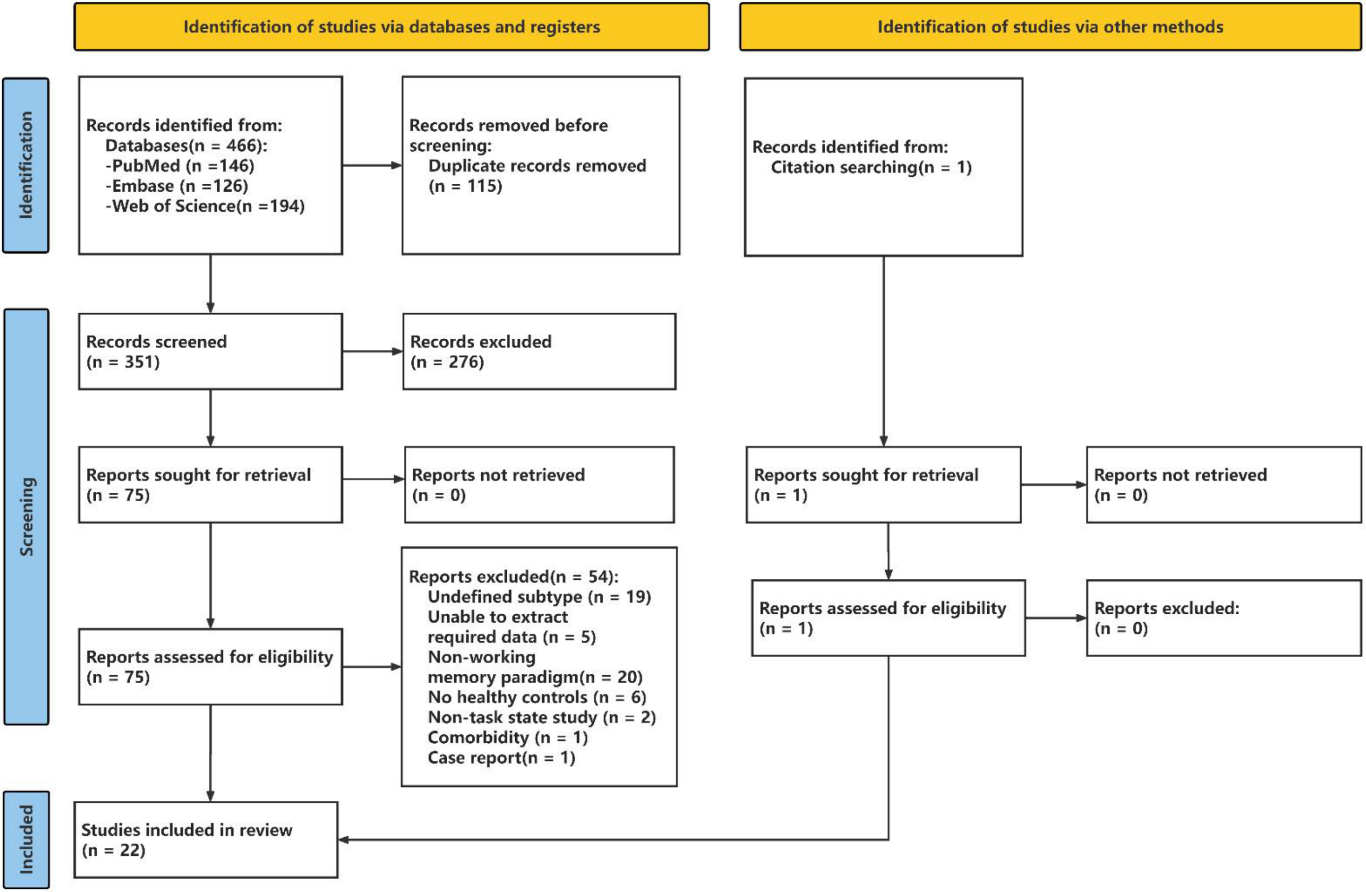
PRISMA flowchart of data searching and selection.

The systematic search of PubMed, Web of Science, and Embase yielded 466 records. After removing 115 duplicates, 351 unique records remained for title/abstract screening. Of these, 276 were excluded because they did not meet the eligibility criteria. Seventy-five full-text articles were retrieved and assessed, and 54 were excluded at the full-text stage. The primary reason for exclusion was failure to specify bipolar subtypes (n = 19), underscoring the frequent conflation of BD subtypes in the literature. Additional reasons for exclusion were the use of non–WM paradigms (n = 20), absence of a healthy control group (n = 6), insufficient or non-extractable data for the outcomes of interest (n = 5), non–task-based fMRI designs (n = 2), significant comorbidity (n = 1), and case reports (n = 1). Reasons were recorded as the primary reason for exclusion for each study. Ultimately, 21 studies met inclusion criteria and were included in the qualitative synthesis, with one additional eligible study identified through reference-list screening, resulting in a total of 22 included studies. The selection process is summarized in the PRISMA flow diagram (**Figure 1**).

### 3.2 Characteristics of included studies

**Supplementary Table 2** summarizes the characteristics and main findings of the included studies. The final selection consisted of 22 task-based fMRI studies published between 2011 and 2025. After accounting for sample overlap across three reports (59–61) derived from the same cohort, the pooled non-overlapping sample comprised 580 participants with BD and 644 healthy controls (HC). Among participants with BD, 529 had bipolar I disorder (BD-I) and 51 had bipolar II disorder (BD-II). With respect to subtype coverage, the literature predominantly included BD-I samples (n = 19); only one study focused exclusively on BD-II (46), and two studies provided direct head-to-head comparisons between BD-I and BD-II (38, 58).

**Figure 2:**
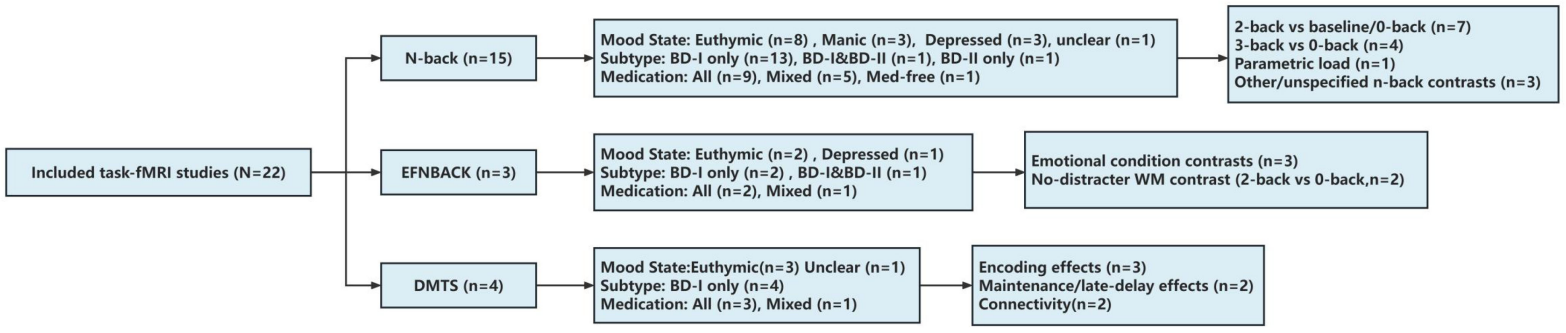
Schematic overview of included task-fMRI studies by paradigm, contrast, and sample characteristics.

Regarding clinical status at the time of scanning, 14 studies recruited euthymic participants (36, 38, 47, 58–63, 67, 69–72), four studies assessed participants during depressive episodes (12, 46, 65, 66) and three studies recruited manic participants (35, 64, 67). Mood-state categories were not mutually exclusive because one study enrolled two (67), and two studies did not map cleanly onto a single mood episode (68, 73). Concerning experimental paradigms, n-back tasks were used in 18 studies (12, 35, 36, 38, 46, 47, 58–69) (15 standard n-back; 3 EFNBACK), and the DMTS task was used in four studies (70–73). Additionally, six studies (58, 64, 68–70, 72) assessed functional or effective connectivity, and one study (66) applied graph-theory metrics to characterize network topology. Notably, BD-II evidence was scarce relative to BD-I, constraining subtype-specific inference. **Figure 2** provides a hierarchical overview of the included task-fMRI studies, organized by working-memory paradigm, primary contrasts/analysis types, and study-level sample characteristics (mood state, subtype coverage, and medication status).

### 3.3 Behavioral performance

Behavioral outcomes were primarily reported as accuracy and reaction time (RT) during WM performance. Across the 22 included studies, 10 reported no significant BD–HC differences in accuracy and/or RT, 11 reported significant BD-related behavioral differences on at least one index/condition (e.g., accuracy, RT, or d′), and one did not provide extractable group-contrast behavioral results.

#### 3.3.1 N-back/EFNBACK

Among N-back studies, Nine studies reported no statistically significant group differences in accuracy/RT (12, 36, 38, 46, 59–61, 64, 69). In contrast, five studies reported statistically significant group differences that were condition-specific: two studies reported load-sensitive reductions in accuracy and/or RT slowing at higher demand levels (66, 68), and three studies reported significantly lower performance across both 1-back and 2-back conditions (35, 63, 65). In addition, subgroup- or state-specific effects were reported in two studies. One study found that manic BD-I participants showed statistically significant differences in accuracy and/or RT compared to HC (67). The other study (47) stratified euthymic BD-I participants into cognitively impaired (CI) and cognitively preserved (CP) subgroups using RBMT (Rivermead Behavioural Memory Test) and BADS (Behavioural Assessment of the Dysexecutive Syndrome) normative 5th-percentile cutoffs (CI: RBMT screening score ≤7 and/or BADS profile score ≤11; CP: RBMT ≥8 and BADS ≥12). In that study, significant behavioral group differences from HC were reported for the CI subgroup in the 1-back and 2-back conditions, whereas the CP subgroup did not show significant differences. Only three studies included BD-II (one BD-II-only study and two BD-I vs. BD-II comparisons) (38, 46, 58). Across these studies, BD-II showed no statistically significant differences from HC on standard n-back tasks, and one direct comparison reported no statistically significant BD-I vs. BD-II behavioral differences (38). In an EFNBACK study, BD-I—but not BD-II—showed statistically significant RT slowing under emotional distraction at 2-back (58).

#### 3.3.2 DMTS

In DMTS paradigms (n=4), one study reported no significant behavioral differences (72). Other studies reported either difficulty-dependent reductions in accuracy (70), slower RT without accuracy differences (73), or overall lower WM accuracy in BD-I relative to HC (71).

### 3.4 Functional fMRI results

Across the 22 included task-based fMRI studies, task-evoked group differences during WM paradigms were most frequently reported in lateral prefrontal and parietal cortices, with reported effects varying across contrast specification, cognitive load, and clinical state.

#### 3.4.1 N-back

In euthymic BD-I cohorts, two studies reported increased activation in right lateral prefrontal regions—such as the middle frontal gyrus/DLPFC and ventrolateral/frontopolar (vlFPC/frontopolar) areas—relative to healthy controls under specific task contrasts (38, 64). Three euthymic BD-I studies reported reduced activation in bilateral middle frontal regions together with increased activation in temporal cortex and/or ACC (59, 61, 62). One euthymic study reported no significant BD-I vs. HC activation differences for its primary contrast (60). One study reported lower right DLPFC activation in a BD-I CI subgroup compared with a CP subgroup (47). In mood-episode samples, two studies reported reduced lateral prefrontal and parietal activation in manic BD-I for 2-back contrasts (35, 67), and one study reported reduced activation in lateral prefrontal and cerebellar regions in depressed BD-I (65). One longitudinal study reported state-related differences between mania and euthymia within BD-I (67). For midline regions reported in terms of deactivation, two studies reported reduced deactivation in medial prefrontal/orbitofrontal and anterior cingulate regions in euthymic BD-I (47, 63), two studies reported reduced deactivation in manic BD-I (35, 64), and one study reported reduced deactivation in depressed BD-I (65). Posterior midline involvement (e.g., PCC and/or precuneus) was reported in two studies during n-back contrasts (65, 68). Studies also reported connectivity and network-level differences during n-back tasks. Within euthymic BD-I, one study reported reduced coordination across DLPFC–parietal–ACC pathways (61). Effective connectivity differences between mPFC and PCC (including differences in direction/sign) were reported in one study with a mixed/unclear mood-state sample (68). One graph-theory study reported group differences in degree centrality across prefrontal/midline and posterior regions with load-dependent patterns involving parietal nodes (66). Regarding BD subtype, one head-to-head comparison reported that BD-II showed an intermediate right lateral prefrontal activation profile that was not statistically different from either BD-I or HC (38). One BD-II-only study (depressed, unmedicated BD-II) reported reduced parametric load-related recruitment across frontal, parietal, temporal/angular, and posterior regions relative to controls (46).

#### 3.4.2 EFNBACK

In EFNBACK paradigms, two studies reported condition-specific effects involving DLPFC/middle frontal regions together with amygdala and striatal responses (58, 69), and one study reported increased putamen activation to face conditions during 2-back in BD-I relative to controls (12). Under emotional interference, one study reported that during fear-related distraction, BD-II showed significantly stronger negative DLPFC–amygdala functional connectivity than both BD-I and HC, while BD-I did not differ significantly from HC (58). One study reported valence-dependent cingulate-to-amygdala effective connectivity differences (69).

#### 3.4.3 DMTS

Within DMTS paradigms, studies reported phase-specific group effects. During the encoding phase, 2 studies reported reduced recruitment of lateral prefrontal regions in BD-I (70, 71), and one study reported attenuated activation within the frontoparietal attention network—including the frontal eye fields (FEF), intraparietal sulcus (IPS), and superior parietal lobule (SPL) (73). During the maintenance phase, one study reported increased activation in posterior/sensory regions (70), whereas one study reported decreased FEF/IPS activation during the late delay period (73). In addition, one euthymic BD-I DMTS study using PPI analysis reported a significantly reduced negative functional connectivity between the right amygdala and the right precentral gyrus, right FEF, right intraparietal cortex (IPS), and (pre-)supplementary motor area (pre-SMA), compared with HC (72).

### 3.5 Medication

Of the 22 included studies, one study recruited a completely medication-free BD sample (46). Seven studies included mixed medicated and unmedicated BD samples (12, 36, 59–61, 66, 72), and 14 studies explicitly reported that all BD participants were medicated during scanning. The most frequently reported medication categories were mood stabilizers (21/22), antipsychotics (20/22), and antidepressants (18/22); benzodiazepines/anxiolytics were mentioned in 10/22 studies (**Supplementary Table 2**).

Medication-related analyses (including *post-hoc*/sensitivity analyses with medication, where reported) were described in 13/22 studies. 12 studies examined medication variables (e.g., medication category and/or dose metrics) in relation to behavioral and/or fMRI outcomes and reported no statistically significant associations with the primary behavioral or fMRI results (12, 35, 36, 58, 59, 61, 62, 65, 66, 68, 71, 73). In contrast, one DMTS study reported statistically significant associations between composite drug load measures and selected task/fMRI measures (70). The remaining 8 studies did not report medication effects in their Results sections (38, 47, 60, 63, 64, 67, 69, 72).

## 4 Discussion

This systematic review synthesizes task-based fMRI evidence on WM in bipolar disorder. The literature is dominated by BD-I samples, with comparatively sparse BD-II coverage. Across paradigms, reported effects vary with contrast specification, cognitive load, mood state, and analytic approach; therefore, we summarize convergent patterns while treating mechanistic interpretations as working hypotheses to be tested in future harmonized, adequately powered studies.

### 4.1 Neural mechanisms of BD-I WM based on the n-back task

#### 4.1.1 CEN: inefficiencies and compensatory mechanisms

A commonly invoked interpretation of WM-related CEN/frontoparietal findings in BD-I is a neural inefficiency account, in which recruitment may vary nonlinearly with cognitive load and clinical state (74). Across the included n-back studies, however, euthymic BD-I results are mixed and appear sensitive to contrast definition, load, and sample characteristics. Thus, rather than indicating a single direction of abnormality, the evidence is most consistent with heterogeneous, state- and demand-dependent variation in frontoparietal recruitment.

During euthymia and under low-to-moderate cognitive load, several studies reported greater activation in lateral prefrontal regions relative to healthy controls (e.g., right middle frontal gyrus/DLPFC) (38, 58) and/or frontopolar/vlFPC regions (36). In parallel, increased activation has been reported in lateral temporal regions (e.g., STG/MTG) in some contrasts (36, 59, 62). Such patterns may be compatible with additional recruitment in at least some cohorts/contrasts, but it remains difficult to distinguish compensation from other sources of between-study variability (e.g., performance matching, medication exposure, and analytic pipelines). Where temporal regions are emphasized, interpretations invoking phonological rehearsal should be treated cautiously but are broadly consistent with evidence linking auditory–temporal pathways to rehearsal-based processes in verbal WM (33, 39). A longitudinal study further reported increased activation in left DLPFC/precentral gyrus as individuals with BD transitioned from mania to euthymia (67), suggesting that recruitment can vary with clinical state.

Under higher load (e.g., 3-back) and/or during acute mood episodes, reduced activation in lateral prefrontal/frontopolar and related regions has been reported in several studies (35, 36, 59, 61, 62, 65, 67). One possibility is that recruitment shows limited scalability under greater demand in at least a subset of cohorts, although this remains provisional given heterogeneity across tasks and contrasts. Individual variability is also suggested by findings of reduced frontoparietal engagement in some euthymic samples even during standard 2-back performance (69). Finally, one study reported that lower DLPFC activation was more evident in a BD-I CI subgroup relative to a CP subgroup (47), indicating that executive recruitment may covary with objective cognitive status.

Overall, the BD-I n-back literature supports load- and state-sensitive variability in frontoparietal recruitment. Mechanistic claims about fixed compensatory thresholds or specific causal pathways should be treated as tentative and prioritized for replication in larger, longitudinal, and methodologically harmonized samples.

#### 4.1.2 DMN: persistent interference and characteristic alterations

In addition to task-positive recruitment differences, the BD-I n-back literature shows a relatively consistent signal of reduced task-related deactivation in midline regions commonly associated with the DMN during WM. Importantly, this pattern has been reported across manic, depressive, and euthymic samples (35, 47, 63–65), is consistent with the possibility that impaired DMN suppression during externally focused WM demands is a recurrent feature of BD-I. Although the exact loci and contrasts differ across studies, the cross-state recurrence strengthens the inference that DMN downregulation is frequently compromised in BD-I during WM.

In manic samples, reduced deactivation has been reported primarily in anterior midline structures (e.g., gyrus rectus, mPFC, frontopolar cortex, vACC) and, in at least one study, extends to medial temporal lobe regions. Posterior midline involvement (PCC and/or precuneus) has also been reported in depressed or mixed samples (65, 68). In euthymia, reduced deactivation in medial prefrontal/orbitofrontal and anterior cingulate regions has been reported (47, 63), indicating that DMN suppression differences can be detectable even outside acute episodes. Notably, a longitudinal study found broadly similar vmPFC deactivation as individuals with BD transitioned from mania to euthymia (67), which adds temporal support to the idea that DMN suppression abnormalities may not be purely state-limited, though further longitudinal work is needed to define their stability and specificity.

From a network-dynamics perspective, insufficient DMN downregulation during WM provides a parsimonious account of how internally oriented processing could intrude on task engagement, increasing the effective background load on executive systems. While methodological diversity (e.g., deactivation contrasts and analytic pipelines) warrants caution against overgeneralization, the available evidence suggests that reduced DMN deactivation may be a candidate marker of WM-related network dysregulation in BD-I and a priority target for replication in harmonized designs.

#### 4.1.3 Dysfunction of auxiliary systems

Beyond CEN and DMN findings, a subset of studies suggests that WM-related alterations in BD-I may also involve additional supportive and affective systems, though the evidence base is more limited and heterogeneous.

Reduced bottom-up support signals. During WM tasks, sensory cortices (e.g., occipital visual cortex) support the primary representation of sensory information (75), and cerebellar circuits have been implicated in temporal prediction, error correction, and sensory–cognitive coordination (76–78). In the included studies, depressed BD-I samples showed attenuated cerebellar recruitment with increasing load (65), and one euthymic study reported reduced activation in cerebellar and occipital regions (63). A graph-theory analysis also reported group differences in degree centrality involving posterior and cerebellar nodes (66). These findings may indicate less robust engagement of sensory–cerebellar support processes under some conditions, which could in turn increase demands on executive control; however, this interpretation requires replication across paradigms and cohorts.

Altered task-state modulation of limbic–striatal systems. Emotional n-back paradigms reported condition-specific limbic/striatal responses alongside prefrontal engagement (12, 58, 69), and reduced deactivation in limbic/medial temporal regions has been reported in manic BD-I (35). In addition, reduced deactivation in caudate has been reported in euthymia (63), and increased activation in putamen/nucleus accumbens has been reported under some task conditions (12, 58, 69). Conceptual frameworks such as basal ganglia gating models (79) provide an interpretive lens for how striatal signals might influence updating and distractor filtering, but the current task-fMRI evidence does not permit strong claims about specific gating parameters (e.g., thresholds). More generally, these observations are consistent with the possibility that affective/reward-related circuitry interacts with WM-related control processes in BD-I, particularly under emotional challenge.

In summary, auxiliary-system findings broaden the picture beyond canonical CEN–DMN interactions, but the limited number of studies and heterogeneous paradigms warrant cautious interpretation.

#### 4.1.4 Multi-level dysregulation of network connectivity coordination

Compared with activation/deactivation findings, the connectivity literature in BD-I is smaller, but the available studies converge on a coherent picture: WM in BD-I is accompanied by altered coordination within control circuits, atypical midline interactions, and context-sensitive emotion–cognition coupling (61, 64, 66, 68). The current limitation is primarily the breadth of evidence (often single studies per approach), rather than an absence of signal.

At the nodal/topological level, a graph-theory study reported load-dependent group differences in degree centrality across prefrontal/midline and posterior regions (66), consistent with demand-sensitive changes in hub-like integration during WM in BD-I. While replication with comparable pipelines is required to establish whether a reproducible “hub shift” occurs, these findings already indicate that network-level reconfiguration under load differs from controls. Interpreting these patterns in terms of parietal–frontal integration is broadly compatible with P-FIT frameworks (80), and provides a plausible organizing account for why WM deficits may not reduce to isolated regional hypo/hyperactivation.

At the circuit level, one euthymic BD-I study reported reduced coordination across DLPFC–parietal–ACC pathways during WM (61), directly implicating frontoparieto-cingulate circuitry that supports maintenance and updating. For midline dynamics, one study reported model-dependent effective connectivity differences between mPFC and PCC in a mixed/unclear mood-state sample (68), highlighting that altered midline regulation can be expressed at the level of directed coupling, although the specificity of effective-connectivity findings depends on model structure.

At the emotion–cognition interface, valence-dependent cingulate-to-amygdala effective connectivity differences have been reported (69), indicating that emotional context meaningfully shapes directed coupling during WM in BD-I. During manic episodes, excessive coupling in frontopolar/superior frontal regions during task states has also been reported (64), consistent with abnormal state-related network engagement, even if links to specific manic cognitive symptoms remain to be clarified.

Overall, the existing connectivity findings support altered integration within control circuits (DLPFC–parietal–ACC), atypical midline interactions, and emotion-dependent coupling in BD-I (61, 64, 66, 68, 69). The priority for the field is replication in larger, harmonized samples to determine which connectivity signatures are robust and state-sensitive.

### 4.2 Neural mechanisms of WM in BD-I based on the DMTS paradigm

The n-back literature demonstrates that recruitment and suppression processes in BD-I vary with load and mood state. DMTS paradigms complement n-back by separating WM into temporally ordered stages (encoding, maintenance, retrieval), allowing more specific inference about when network-level disruption emerges. Although DMTS evidence in BD-I is based on a small number of studies (predominantly euthymic samples), the existing findings outline a consistent stage-resolved profile that links early task initiation to later maintenance strategies.

#### 4.2.1 Encoding phase: impaired network initiation and cascading disruption in recruitment

Encoding depends on rapid allocation of cognitive resources and task-state configuration, processes commonly attributed to the SN (18). In a DMTS study, euthymic BD-I participants showed reduced encoding-related activation in bilateral insula, thalamus, and striatum (70). Given the anterior insula’s established role in switching between internally oriented and task-focused network states (18, 19, 81), this pattern is consistent with inefficient task-state switching at task onset, which would plausibly undermine stable engagement of downstream control systems during encoding.

Consistent with this, DMTS studies also report attenuated encoding-related recruitment in lateral prefrontal regions (70, 71) and reduced engagement of DAN hubs (FEF, IPS) (73). Taken together, the DMTS encoding evidence points to a coordinated disturbance spanning SN-linked switching signals, executive recruitment, and attention allocation (70, 71, 73). A key next step is to test whether these encoding-phase differences predict trial-level encoding accuracy and downstream maintenance stability.

#### 4.2.2 Maintenance phase: reduced top-down maintenance strategies and low-level sensory compensation

During the maintenance interval, DMTS findings suggest that attentional control signals can remain attenuated over time. One study reported reduced activation in FEF and IPS during the late delay period (73), indicating weaker sustained engagement of attention/control nodes during maintenance.

In contrast, one study reported increased delay-period activation in posterior/sensory regions (bilateral cuneus, middle occipital gyrus, right postcentral gyrus) in BD-I (70). This pattern aligns with sensory recruitment accounts (82), in which maintenance relies more heavily on perceptual/imagery-based representations when higher-order top-down signals are less stable. Across studies, the emerging picture is that BD-I maintenance may reflect a shift in strategy or resource allocation—from sustained frontoparietal attentional maintenance toward greater reliance on posterior representations—whose functional consequences for load sensitivity and distractor resistance should be tested directly.

#### 4.2.3 Network dedifferentiation and the intrusion of affective interference

Beyond stage-specific activation changes, DMTS studies also implicate altered network coupling that points to reduced segregation between midline/affective systems and task networks. One study reported increased positive functional connectivity between mPFC and right IFG during WM (70), consistent with reduced functional segregation between midline regions and control circuitry. Under typical conditions, DMN activity is suppressed and remains relatively segregated from task-positive control networks during externally oriented tasks (17); therefore, increased mPFC–IFG coupling during WM is compatible with diminished separation between internal and task-focused processing streams.

In addition, a PPI study reported reduced negative coupling between the amygdala and motor/attention-related regions (including precentral gyrus, FEF, IPS, and pre-/SMA) (72), consistent with weaker limbic-to-task network separation during WM. Together, these coupling patterns support a model in which BD-I WM is characterized not only by altered recruitment across phases, but also by reduced network segregation that could increase susceptibility to affective contextual signals during task performance (70, 72).

### 4.3 Neural mechanistic characteristics of WM in BD-II during the n-back task

Relative to BD-I, BD-II evidence remains limited, but the available studies support a more state- and context-dependent profile rather than a stable, generalized deficit. Across the small BD-II literature, neural differences emerge most clearly when WM is challenged by depressive state and/or higher demand, while euthymic performance and recruitment can be relatively preserved.

At the level of the CEN, euthymic BD-II shows an intermediate lateral prefrontal activation profile that does not significantly differ from controls in one direct comparison (38), and available studies report broadly comparable behavioral performance to controls under some conditions (38, 58). This pattern supports the view that BD-II can sustain WM engagement during euthymia without the more pronounced recruitment instability often described in BD-I.

However, during depressive episodes, a BD-II-only study reported a load-dependent upregulation deficit across lateral prefrontal, parietal, and posterior association regions as WM demand increased (46). In that study, BD-II depression was associated with reduced load-related upregulation as task demand increased.

Regarding DMN-related findings, the limited BD-II literature has not yet provided consistent evidence for a BD-I–like cross-state pattern of incomplete task-related DMN deactivation. Instead, the depressed BD-II study reported insufficient load-related modulation in regions adjacent to the DMN (angular gyrus, precuneus) (46), which may reflect a narrower dynamic range of task-related modulation rather than a broadly generalized pattern of reduced deactivation.

Finally, affective context appears to differentiate coupling patterns. Under fear interference, BD-II showed stronger negative DLPFC–amygdala functional connectivity than BD-I and controls (58). While this result currently rests on a single paradigm, it is consistent with a more constrained or actively regulated limbic response during threat-related distraction in BD-II.

### 4.4 Differentiating WM network abnormalities in BD-I vs BD-II: preliminary evidence for cross-state inefficiency and state-/context-dependent recruitment changes

Synthesizing the available n-back evidence (and limited DMTS findings in BD-I), the current qualitative literature suggests that BD-I and BD-II may show partially divergent profiles, although inferences about BD-II remain constrained by the small and heterogeneous evidence base (38, 46, 58).

At the level of the CEN, BD-I findings are often interpreted within a neural inefficiency/limited scalability framework (74). During euthymia and under low-to-moderate load, studies report either increased lateral prefrontal engagement or mixed frontoparietal patterns (sometimes extending to temporal regions) while performance is relatively preserved (38, 58, 59, 61, 62, 64). Under higher load and/or acute manic/depressive episodes, reduced engagement of lateral prefrontal/frontopolar and related regions has been repeatedly reported (35, 36, 59, 61, 62, 65, 67), which is consistent with the possibility that recruitment becomes less scalable as demand increases in at least a subset of cohorts. In contrast, in the limited BD-II evidence, euthymic BD-II more often shows near-normative executive recruitment and behavioral performance (38, 58), with abnormalities most clearly reported during depressive states as attenuated load-related upregulation and reduced dynamic range (46)—consistent with a state-dependent recruitment vulnerability account.

At the level of the DMN, BD-I is more consistently characterized by reduced task-related deactivation across manic, depressive, and euthymic states (35, 47, 63–65), raising the possibility of relatively persistent background interference from internally oriented processing during WM demands in some individuals. Longitudinal evidence provides preliminary support for persistence in vmPFC deactivation across mania-to-euthymia transition (67), although additional longitudinal work is required before treating this as a definitive trait marker. In BD-II, current evidence is insufficient to support a comparable cross-state deactivation phenotype; DMN-related abnormalities are currently limited to reduced load-related modulation in a depressed BD-II sample (46), which may reflect reduced modulatory capacity under state-dependent conditions.

Regarding affective–cognitive interactions, subtype differentiation is suggested but remains based on a small evidence base. In BD-I, emotional n-back findings include condition-specific limbic/striatal responses alongside prefrontal engagement (12, 58, 69) and valence-dependent cingulate-to-amygdala effective connectivity differences (69). In BD-II, stronger negative DLPFC–amygdala coupling under fear distraction has been reported in one paradigm (58), which may be consistent with a distinct prefrontal–limbic coupling pattern during threat interference. Under positive conditions within the same paradigm, BD-II showed a comparatively constrained subcortical response profile relative to BD-I (58), though replication is needed before drawing firm conclusions about defensive gating.

**Figure 3:**
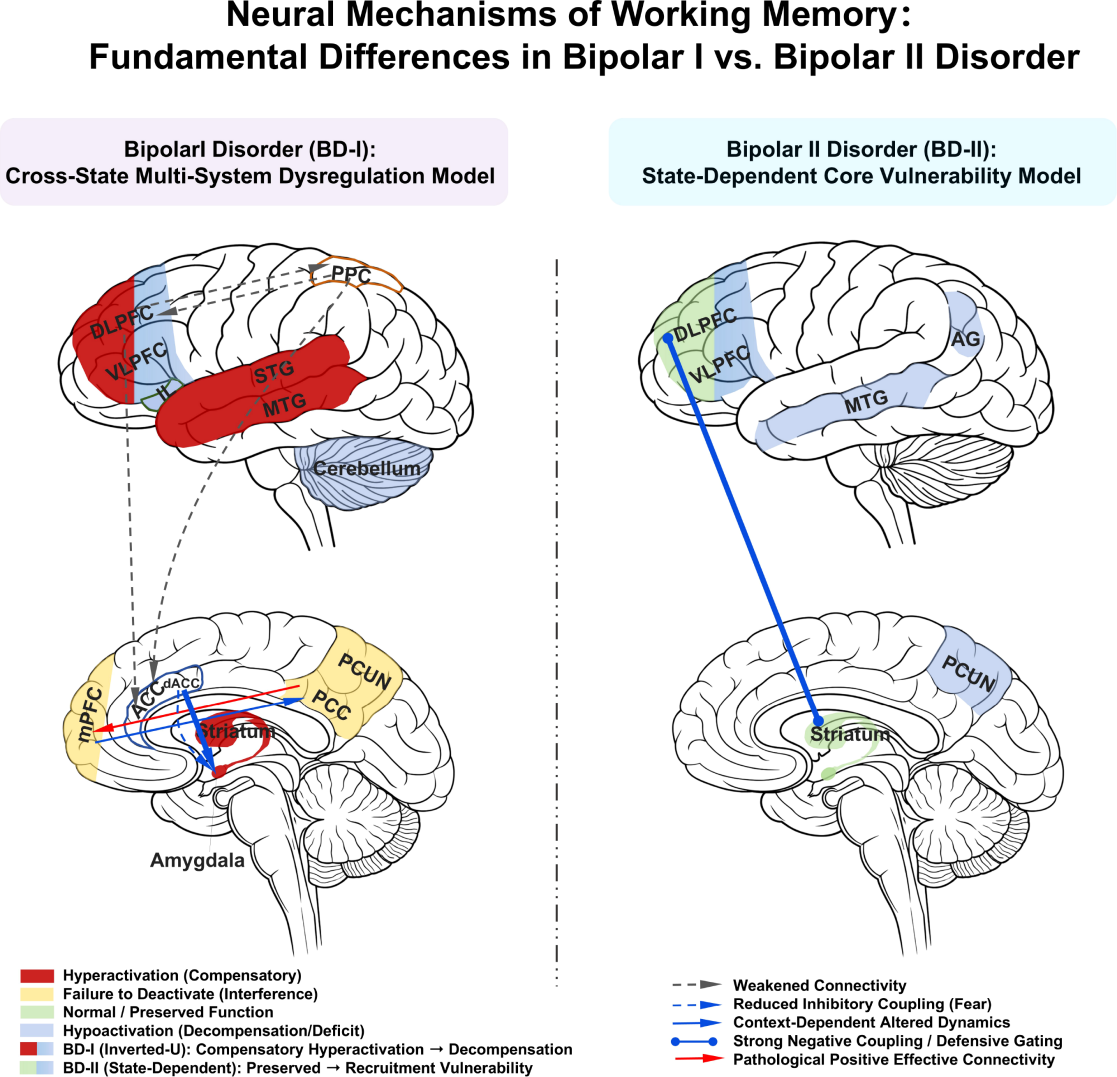
Putative neural mechanisms of WM in BD-I vs BD-II (conceptual schematic based on qualitative evidence).

Finally, DMTS findings provide stage-resolved evidence that BD-I differences can emerge early in processing: reduced recruitment of insula/thalamus/striatal regions and lateral prefrontal/attention systems during encoding (70, 71, 73), alongside altered limbic-to-task coupling during WM (72). In BD-II, evidence for stage-specific mechanisms remains limited. Overall, BD-I appears more consistent with multisystem alterations across mood states and reduced network differentiation, whereas BD-II is currently better characterized by state-dependent recruitment bottlenecks with putative differences in prefrontal–limbic coupling under negative distraction (**Figure 3**).

BD-I: Cross-state multi-system dysregulation. This schematic summarizes a pattern that has often been interpreted as relatively persistent neural inefficiency, illustrated by the red/blue inverted-U gradient in the DLPFC (putative compensatory hyperactivation at lower load with reduced recruitment at higher demand), together with reduced task-related DMN suppression/interference (yellow). Connectivity lines (gray/blue dashed and red arrows) are intended to schematically reflect reported alterations in within- and between-network coupling (e.g., reduced segregation and/or altered synchronization), rather than implying a specific causal mechanism. BD-II: State-dependent core vulnerability. Based on a limited evidence base, the schematic illustrates reports suggesting that executive recruitment and performance may be relatively preserved in euthymia (green) but may show reduced scalability or recruitment vulnerability under higher load and/or depressive states (blue). A potential differentiating observation from one emotional-interference paradigm is stronger negative DLPFC–amygdala coupling under threat distraction (solid blue dumbbell line), which has been interpreted as consistent with a defensive gating account; however, this interpretation remains provisional and requires replication. Colors and connection lines are illustrative and do not represent quantitative effect sizes. Abbreviations: ACC, anterior cingulate cortex; AG, angular gyrus; DLPFC, dorsolateral prefrontal cortex; IL, Insular Lobe; mPFC, medial prefrontal cortex; MTG, middle temporal gyrus; PCC, posterior cingulate cortex; PCUN, precuneus; PPC, posterior parietal cortex; STG, superior temporal gyrus; VLPFC, ventrolateral prefrontal cortex.

After completion of our search, a closely related systematic review by Mardani (83) was published, synthesizing task-based fMRI studies of memory and attention in BD that explicitly linked in-scanner task activation to out-of-scanner neuropsychological performance. Across 10 eligible studies, Mardani et al. reported that poorer working memory and executive functioning were associated with reduced recruitment of prefrontal regions (including inferior frontal and ventrolateral/dorsolateral prefrontal cortices), alongside reduced task-related suppression of default-mode regions, supporting the notion that impaired cognitive performance in BD is reflected in altered engagement of prefrontal control systems and insufficient DMN downregulation. While their review primarily addressed brain–behavior correlations across memory/attention domains, the present review is complementary in scope by focusing specifically on working-memory task fMRI and synthesizing findings at the network level, with particular attention to subtype-related patterns (BD-I vs BD-II) and study quality considerations using a dedicated fMRI methodological checklist. Taken together, both reviews converge on the central role of prefrontal control circuitry and DMN interference in BD-related cognitive dysfunction, while highlighting the need for larger, better-controlled studies to support subtype-specific inferences.

## 5 Limitations

While this review aims to delineate WM–related neural mechanisms that may differentiate BD-I from BD-II, the current evidence base warrants cautious interpretation. First, our *a priori* restriction to studies published from 2011 onward may have excluded earlier task-fMRI WM studies in bipolar disorder, particularly those that did not report subtype-stratified results; therefore, conclusions should be interpreted as pertaining to the contemporary subtype-reporting literature. Second, studies that directly compare BD-I and BD-II within a unified experimental framework remain scarce. Consequently, the putative subtype-specific patterns are disproportionately driven by a limited number of pivotal reports, and their external validity and robustness require replication in larger, independent cohorts and multi-center datasets. By contrast, the majority of available investigations contrast either BD-I or BD-II with healthy controls. Although these studies have advanced our understanding of large-scale network dysfunction, they provide only indirect support for subtype differentiation and are more vulnerable to heterogeneity in sampling, task parameters, and analytic pipelines. Moreover, this prevalent design limits the capacity to determine whether observed alterations reflect features specific to BD versus transdiagnostic characteristics of affective psychopathology more broadly, as comparable abnormalities may also be present in other populations (e.g., MDD) in the absence of direct cross-diagnostic comparisons.

Third, subtype contrasts are highly contingent upon mood state and broader clinical heterogeneity. Included studies span euthymic, depressive, and manic/hypomanic phases, and the recruitment of WM–relevant networks (CEN/DMN/SN/DAN), the intensity of inter-network competition, and affect–cognition coupling may differ substantially across states. Without rigorous matching of BD-I and BD-II groups on mood state, illness phase, lifetime psychotic features, and other clinically salient characteristics, apparent subtype differences may reflect state-dependent effects or residual confounding by clinical covariates rather than trait-like neurobiological distinctions.

Fourth, medication exposure is heterogeneous across studies and is frequently insufficiently characterized or controlled. Most samples comprise medicated individuals or mixed medicated/unmedicated cohorts. Even among the few direct BD-I versus BD-II comparisons, subtype groups often differ in the prevalence and combinations of antipsychotics, mood stabilizers, and antidepressants. Moreover, statistical control is commonly limited to coarse medication categories, without quantifying dose equivalence, overall medication load, or cumulative treatment history. Given the capacity of psychotropic agents to modulate BOLD activation and connectivity indices in network- and task-dependent ways, medication effects may either exaggerate or mask genuine subtype-related neural differences.

Heterogeneity in methods also limits what can be concluded across studies. Paradigms and contrast definitions vary (e.g., N-back vs DMTS; 2-back vs 0-back/baseline), and these choices can meaningfully change the processes being taxed and the networks that appear engaged. Analytical decisions differ as well—ROI versus whole-brain approaches, correction thresholds, and model structure—making direct comparison difficult. Moreover, PPI, functional connectivity, and effective connectivity are not interchangeable; they index related but distinct aspects of inter-regional coupling.

Future work will be more informative if BD-I and BD-II are examined within the same protocol and at adequate sample sizes. Clearer reporting of load manipulations and dose–response patterns across levels of WM demand would help. Attention to mood state, illness phase, and medication exposure—through matching, stratification, or explicit covariate modeling—should reduce avoidable confounding. Multimodal imaging may then offer convergent tests of whether apparent subtype differences persist across measurement levels.

## 6 Conclusion

Drawing on the available task-based fMRI literature using WM paradigms, the qualitative evidence suggests that BD-I and BD-II may show partially different profiles of large-scale network engagement during WM processing; however, these inferences are constrained by the small number of included studies overall and the particularly limited BD-II evidence base. In the small set of studies that directly contrasted the two subtypes, BD-II under neutral, non-emotional WM demands was often reported to show behavioral performance and cortical activation closer to healthy controls, or intermediate between BD-I and controls, although results varied across paradigms and samples. By contrast, BD-I studies more frequently reported patterns that have been interpreted as less efficient or less scalable recruitment of executive-control resources as task demands increased, including indications of putative compensatory activation at lower load that did not consistently extend to higher demand conditions.

Under higher WM load and/or emotional interference, BD-I studies more often reported reduced CEN recruitment and incomplete task-related suppression of DMN regions. These observations are compatible with (but do not establish) the possibility of greater competition from internally oriented processes during externally oriented task states. For BD-II, the available literature does not yet support a simple less severe BD-I characterization across contexts. Instead, the limited evidence to date suggests that BD-II findings may be more state- and context-dependent: during emotionally salient interference—particularly threat-related distraction—some studies have reported relatively preserved performance together with increased prefrontal involvement and more negative prefrontal–amygdala coupling. Importantly, the BD-II literature remains sparse, and additional adequately powered, direct BD-I/BD-II comparisons are needed before treating these patterns as robust or subtype-specific.

Accordingly, the subtype-differentiation account advanced here is presented as a testable working model intended to guide future direct-comparison studies rather than as a definitive conclusion. Future work using harmonized paradigms with sufficient sample sizes—ideally stratified by mood state and incorporating dynamic functional connectivity and multimodal imaging—will be better positioned to evaluate whether putative subtype differences relate primarily to: (1) the degree of task-related DMN suppression and potential competition from endogenous mentation; (2) the scalability of CEN up-regulation with increasing load and possible limits on compensatory recruitment; and/or (3) context-dependent emotion-related modulation linking prefrontal control systems with limbic circuitry. If supported, these lines of investigation could help refine subtype-informed hypotheses for cognitive remediation and emotion-regulation interventions and inform the search for candidate neuroimaging markers, while acknowledging that clinical translation will require replication and prospective validation.

## Data Availability

The original contributions presented in the study are included in the article/**Supplementary Material**, further inquiries can be directed to the corresponding author/s.
