# Supplementary material for: Working memory network dysfunction in bipolar I vs. bipolar II disorder: a systematic review of task-fMRI evidence
Source: Front Psychiatry. 2026 Jun 16;17:1800042. doi: 10.3389/fpsyt.2026.1800042 (PMC13320638; doi:10.3389/fpsyt.2026.1800042)
Supplement: Supplementary file 1 [file Table1.docx]

**Supplementary Table S1. Full search strategies and search metadata**

Search strategies are reported exactly as executed on the stated dates; record counts correspond to database results prior to deduplication. Date limits reflect publication date restrictions (01/01/2011-30/06/2025).

## PubMed (NCBI PubMed)

| Database | PubMed |
| --- | --- |
| Platform/Interface | NCBI PubMed |
| Search run date (DD/MM/YYYY) | 12/10/2025 (12 October 2025) |
| Date limits applied (publication date) | 01/01/2011-30/06/2025 |
| Records retrieved (n) prior to deduplication | 146 |
| Full search strategy (exact string as executed) | ("Bipolar Disorder"[MeSH] OR "bipolar i"[tiab] OR "bipolar ii"[tiab] OR "bipolar 1"[tiab] OR "bipolar 2"[tiab] OR "bd-i"[tiab] OR "bd-ii"[tiab] OR "bipolar type i"[tiab] OR "bipolar type ii"[tiab] OR "bipolar I disorder"[tiab] OR "bipolar II disorder"[tiab] OR "manic depression"[tiab] OR "manic-depressive illness"[tiab]) AND ("Memory, Short-Term"[MeSH] OR "Working Memory"[tiab] OR "WM"[tiab] OR "n-back"[tiab] OR "n back"[tiab] OR "nback"[tiab] OR "Sternberg"[tiab] OR "delayed matching to sample"[tiab] OR "DMTS"[tiab] OR "delayed response task"[tiab] OR "spatial working memory"[tiab] OR "verbal working memory"[tiab]) AND ("Functional Neuroimaging"[MeSH] OR "Magnetic Resonance Imaging"[MeSH] OR "fMRI"[tiab] OR "functional MRI"[tiab] OR "task-based fMRI"[tiab] OR "task fMRI"[tiab] OR "functional magnetic resonance"[tiab] OR "BOLD"[tiab] OR "brain activation"[tiab] OR "neural activation"[tiab]) |

## Embase (Embase.com, Elsevier)

| Database | Embase |
| --- | --- |
| Platform/Interface | Embase.com (Elsevier) |
| Search run date (DD/MM/YYYY) | 12/10/2025 (12 October 2025) |
| Date limits applied (publication date) | 01/01/2011-30/06/2025 |
| Records retrieved (n) prior to deduplication | 126 |
| Full search strategy (exact string as executed) | ('bipolar disorder'/exp OR 'bipolar i disorder'/exp OR 'bipolar ii disorder'/exp OR (bipolar NEAR/2 (i OR ii OR 1 OR 2 OR 'type i' OR 'type ii')):ti,ab OR 'bipolar I disorder':ti,ab OR 'bipolar II disorder':ti,ab OR 'bipolar i':ti,ab OR 'bipolar ii':ti,ab OR 'bipolar 1':ti,ab OR 'bipolar 2':ti,ab OR 'bd-i':ti,ab OR 'bd-ii':ti,ab OR 'manic depression':ti,ab OR 'manic-depressive illness':ti,ab) AND ('working memory'/exp OR 'short term memory'/exp OR 'n back task'/exp OR ('working memory' OR wm OR 'short-term memory' OR 'short term memory' OR 'n-back' OR 'n back' OR nback OR sternberg OR 'delayed matching to sample' OR dmts OR 'delayed response task' OR 'spatial working memory' OR 'verbal working memory'):ti,ab) AND ('functional magnetic resonance imaging'/exp OR 'magnetic resonance imaging'/exp OR (fmri OR 'functional mri' OR 'task-based fmri' OR 'task fmri' OR 'functional magnetic resonance' OR bold OR 'brain activation' OR 'neural activation'):ti,ab) |

## Web of Science (Web of Science Core Collection, Clarivate)

| Database | Web of Science Core Collection |
| --- | --- |
| Platform/Interface | Clarivate Web of Science |
| Search run date (DD/MM/YYYY) | 12/10/2025 (12 October 2025) |
| Date limits applied (publication date) | 01/01/2011-30/06/2025 |
| Records retrieved (n) prior to deduplication | 194 |
| Full search strategy (exact string as executed) | TS=("bipolar disorder" OR "bipolar i" OR "bipolar ii" OR "bipolar 1" OR "bipolar 2" OR "bd-i" OR "bd-ii" OR "bipolar type i" OR "bipolar type ii" OR "bipolar I disorder" OR "bipolar II disorder" OR "manic depression" OR "manic-depressive illness") AND TS=("working memory" OR WM OR "short-term memory" OR "short term memory" OR "n-back" OR "n back" OR nback OR Sternberg OR "delayed matching to sample" OR DMTS OR "delayed response task" OR "spatial working memory" OR "verbal working memory") AND TS=(fMRI OR "functional MRI" OR "task-based fMRI" OR "task fMRI" OR "functional magnetic resonance" OR BOLD OR "brain activation" OR "neural activation") |
