# Supplementary material for: Working memory network dysfunction in bipolar I vs. bipolar II disorder: a systematic review of task-fMRI evidence
Source: Front Psychiatry. 2026 Jun 16;17:1800042. doi: 10.3389/fpsyt.2026.1800042 (PMC13320638; doi:10.3389/fpsyt.2026.1800042)
Supplement: Supplementary file 2 [file Table2.docx]

**Supplementary Table 2 Characteristics and results of studies examining neural differences in working memory between Bipolar Disorder I and II**

| **Author and Year** | **Group(n)** | **BD/HC Age,M(SD)** | **BD/HC Female(%)** | **Medications（BD）** | **Emotional state（BD）** | **Task** | **Task Results** | **fMRI Results** | NOS Score | fMRI Tech Score(Table 3) | Quality Level |
| --- | --- | --- | --- | --- | --- | --- | --- | --- | --- | --- | --- |
| Caseras et al., 2015 | BD-I:16, BD-II:19 / HC:19 | 42.56(7.47), 38.74(8.07) / HC:42.30(5.99) | 62.5%, 68.4%/65.0% | All medicated. BD-I (n=16): antipsychotics n=11, mood stabilizers n=7, antidepres sants n=10; BD-II (n=19): antipsychotics n=9, mood stabilizers n=10, antidepressants n=6. The potential impact of medication was analyzed and was not found to explain the primary results. | Euthymic | EFNBACK | BD-I: Significantly slower RT to targets during 2-back with emotional (fear/happy) distractors vs. HC and BD-II. BD-II: RT did not differ from HC. No slowing due to distractors. | 2-back vs 0-back(no-distracters): BD-I showed greater activity within the working memory network(middle frontal gyrus,DLPFC) compare to HC and BD-II. 2-back fear:BD-I and BD-II was observed increased activity the DLPFC,amygdala,and nucleus accumbens compared to HC, BD-II showed greater increased activity than BD-I in the DLPFC and amygdala. 2-back happy: BD-I showed significantly increased activity in the DLPFC, amygdala, and nucleus accumbens compared to HC and BD-II,BD-II showed higher activity than HC only in the amygdala. 2-back neutral: BD-I showed lower DLPFC activity and higher amygdala and nucleus accumbens activity compared to HC. Functional Connectivity (PPI Analysis):In response to fear-related interference,BD-II showed significantly stronger bilateral DLPFC-amygdala negative functional connectivity compared to BD-I and HC. BD-I showed no difference from HC. | 9★ | 4 | High |
| Dell'Osso et al., 2015 | BD-I:15, BD-II:13 / HC:27 | 34.3(10.3),37.3(8.0)/29.4(10.6) | 46.6%, 46.2% / 44.5% | All medicated. BD-I (n=15): MS alone n=10, SSRI n=1, MS+SSRI n=1, MS+other ADs n=2; BD-II (n=13): MS alone n=5, SSRI n=2, MS+SSRI n=3, MS+SNRI n=3.The impact of the drug was not explicitly reported in Results section. | Euthymic | N-back | All groups (BD-I, BD-II, HC) showed no significant differences in task accuracy or reaction time. | BD-I patients had significantly greater activation in the right middle frontal gyrus（DLPFC，BA10） compared to HCs. BD-II patients showed an intermediate pattern of activation between BD-I and HC, not statistically different from either. No significant interaction between diagnosis and WM load was found. | 7★ | 5 | High |
| Brooks et al., 2015 | BD-II:19/HC:19 | 36.70(11.40)/42.60(12.00) | 42.1% / 52.6% | Unmedicated | Depressed | N-back | No significant group differences in accuracy and reaction time.. | Whole-brain results (the parametric analysis of memory load): BD-II showed significantly reduced activity relative to HC in the left middle frontal gyrus (BA11), left superior frontal gyrus (BA10), left inferior parietal lobule (BA40), left middle temporal gyrus and angular gyrus (both BA39), and occipital regions (including precuneus, BA7/19). | 8★ | 5 | High |
| Alonso-Lana et al., 2016 | BD-I(cognitively preserved,CP):27,BD-I(cognitively impaired,CI):23/HC:28 | 44.13(6.63),46.17(7.40)/44.01(6.03) | 45.5%,39.3%/57.1% | All medicated. BD-I CP : MS n=28 (Li alone n=13, other MS n=15), AP n=21 (SGA n=21, FGA n=2), AD n=8; BD-I CI : MS n=28 (Li alone n=13, other MS alone n=6, Li+other MS n=9) The impact of the drug was not explicitly reported in Results section. | Euthymic | N-back | In 1-back and 2-back tasks,only the difference between the CI group and the HC group was statistically significant. | 2-back vs baseline:BD-I CP showed failure of deactivation in the medial prefrontal cortex affecting the gyrus rectus and extended to the medial orbitofrontal and anterior cingulate cortex (peak in BA11) compared to HC. 2-back vs 1-back:BD-I CP showed failure of deactivation in mPFC (peak in BA25) compared to HC.Additionally, BD-I CI showed reduced activation in the right DLPFC (peak in BA8) compared to BD-I CP. | 8★ | 5 | High |
| Delvecchio et al., 2015 | BD-I:41 / HC:46 | 44.3(11.9)/40.3(13.2) | 51.2% / 45.7% | 30 medicated: MS n=21 (Li n=15, Val n=6), AP n=12 (SGA n=7, FGA n=2, both n=3), SSRI n=13; 11 unmedicated.No correlation between brain activation signals and drug dosage was found in the patients | Euthymic | N-back | No significant differences in accuracy and reaction time between groups. | 3-back vs 0-back: Brain activation in BD patients was significantly reduced in the left and right middle frontal gyri (BA9, BA10) and increased in the superior and middle temporal gyri on the right (BA21/22) and in the ACC bilaterally (BA24/32) compared to HC. | 8★ | 4 | High |
| Dima et al., 2016a | BD-I: 41 / HC: 46 | 44.3(11.9)/40.3(13.2) | 51.2% / 45.7% | 30 medicated: MS n=21 (Li n=15, Val n=6), AP n=12 (SGA n=7, FGA n=2, both n=3), SSRI n=13; 11 unmedicated.The impact of the drug was not explicitly reported in Results section.. | Euthymic | N-back | No significant differences in performance between BD and HC on the 2-back task. | 2-back vs baseline:No significant differences in brain activation were found between BD-I and HC. | 8★ | 4 | High |
| Dima et al., 2016b | BD-I: 41 / HC: 46 | 44.3(11.9) / 40.3(13.2) | 51.2% /45.7% | 30 medicated: MS n=21 (Li n=15, Val n=6), AP n=12 (SGA n=7, FGA n=2, both n=3), SSRI n=13; 11 unmedicated.The type and dosage of the drug had no significant effect on behavioral performance and all fMRI results | Euthymic | N-back | No significant differences in accuracy or response time between BD patients and HC. | 3-back vs 0-back:Brain activation in BD-I paients was significantly reduced in the left and right middle frontal gyri(BA9) and increased in the right temporal gyrus(BA21) and bilateral ACC(24/32) bilaterally compared to HC. Widespread WM network hypocomectivity in BD-I involved:Reduced left-right DLPFC reciprocity. Weakened forward (parietal cortex (PAR) →ACC, PAR→DLPFC) and backward (DLPFC→ACC, DLPFC→PAR) pathways. | 8★ | 5 | High |
| Frangou et al., 2017 | BD-I: 30 / HC: 30 | 34.7(7.7)/33.4(11.6) | 50% / 50% | All medicated: AP (n=21), antiepileptics (n=8), Li (n=14); 18 on monotherapy, 12 on polytherapy.The drug status and dosage had no significant effect on brain activation and classification results | Euthymic | N-back | The study did not emphasize direct performance differences between BD patients and HC. | 3-back vs 0-back: BD-I patients showed reduced activation in the middle frontal gyrus (BA10) and increased activation in the superior temporal gyrus (BA22) compared to HC. A Gaussian Process Classifier (GPC) using this contrast discriminated BD from HC with 83.5% accuracy; discriminating regions included left inferior/middle/superior frontal gyri and superior parietal lobule. | 8★ | 5 | High |
| Verdolini et al., 2023 | BD-I: 31 / HC: 31 | 45.54 (9.4) / 44.84 (9.4) | 64.5%/64.5% | All (n=31) on MS, 18 on atypical AP, 8 on AD.The impact of the drug was not explicitly reported in Results section.. | Euthymic | N-back | BD patients performed worse than HC on both 1-back and 2-back tasks. | 2-back vs baseline: BD-I showed a significant failure to deactivate in the bilateral medial frontal cortex and the ACC (BA11), and the left caudate compared to HC. 2-back vs 1-back: Compared to HC,BD-I exhibited not only a significant failure to deactivate in the bilateral medial frontal cortex and ACC（BA11）, and left caudate, but also significantly reduced activation in the cerebellum and occipital region. | 8★ | 5 | High |
| Jogia et al., 2012 | BD-I:36/HC:37 | 42.5(10.6)/37.6(11.3) | 52.8%/43.2% | 22 medicated: MS n=21 (Li), AP n=6, SSRI n=7;14 unmedicated.Drug analysis showed that there was no significant difference in brain activation between the medication group and the non-medication group, and there was no correlation between the dosage of medication and brain activation. | Euthymic | N-back | No significant differences between BD-I and HC. | 3-back vs 0-back: BD-I patients showed reduced activation in the right vlFPC (BA10) and increased activation in the right superior (BA22) and middle temporal gyri (BA21) compared to HC. 2-back vs 0-back: In the same right vlFPC region (BA10), BD-I patients showed increased activation compared to HC. | 8★ | 5 | High |
| Pomarol-Clotet et al., 2011 | BD-I : 29 / HC: 46 | 40.79 (12.08) / 36.28 (13.62) | 37.9%/ 41.3% | All medicated:Mood Stabilizers (n=23: Lithium 16, Valproate 3, Carbamazepine 1, Lamotrigine 1, Lithium+Lamotrigine 2; Antipsychotics (n=24:Atypical 14, Typical 4, Combined 6). No significant effect of medication treatment was observed on brain activation or deactivation patterns in patients. | Mania | N-back | BD-I Patients performed significantly worse than healthy controls on both the 1-back and 2-back conditions. | 2-back vs Baseline: Compared to HC,BD-I patients showed significantly reduced activation in the bilateral DLPFC(BA6)and the right superior parietal cortex(BA7);they also exhibited a significant less deactivation in the the gyrus rectus bilaterally, related frontomedial  structures and the anterior cingulate gyrus(BA10),right temporal pole included the parahip pocampal region, the amygdala and marginally the  hippocampus(BA20),and the left temporal pole included the parahippocampal region and the amygdala(BA38). | 8★ | 5 | High |
| Goikolea et al., 2019 | BD-I :31 / HC:31 | 30.52(9.05)/31.06(8.76) | 48.4% /48.4% | All medicated:AP (n=30), Li (n=14), Val (n=8), BZD (n=8).The impact of the drug was not explicitly reported in Results section. | Mania | N-back | No significant differences in performance between BD and HC groups for the 2-back task. | 2-back vs baseline:BD patients showed less deactivation (failure to deactivate) in a left mPFC cluster encompassing the anterior cingulate cortex (ACC, BA32) and medial frontopolar cortex (FPC, BA10) compared to HC. PPI(2-back):BD-I patients showed increased functional connectivity during the 2-back task between this left FPC seed region and the superior frontal gyrus (BA8) compared to HC. | 9★ | 5 | High |
| Rodríguez-Cano et al., 2017 | BD-I: 26 / HC: 26 | 45.58(9.23)/46.77(11.18) | 61.5% / 61.5% | All medicated: MS n=23 (Li n=15, Val n=6, Lam n=1), AD n=15, AP n=14, Anxiolytics n=24. Analysis showed no significant effect of antipsychotic or antidepressant medication on mPFC activation. | Depressed | N-back | The BD-I group performed significantly worse than the HC group on both the 1-back and 2-back conditions. | 2-back vs Baseline: BD-I showed significantly reduced activation in the left DLPFCand right cerebellum(BA48).They also showed a significant failure to deactivate the mPFC (BA11) compared to HC. ROI Analysis showed that the BD-I group demonstrated significantly greater failure to deactivate in the mPFC(BA11) compared to the HC. 1-back vs Baseline: BD-I showed less deactivation compared to HC in the dmPFC(BA10) and precuneus(BA18). | 8★ | 5 | High |
| Xi et al., 2023 | BD-I:31/HC: 80 | 24.74(5.33)/23.46(4.93) | 61.3% / 52.5% | 30 medicated:MS n=14, AP n=16, AD n=21,BZD n=6. 1 unmedicated. Relevant analysis showed that there was no significant association between the drug dosage and the DC. | Depressed | N-back | BD-I had significantly lower accuracy than HC for the 2-back load. BD-I also had significantly longer reaction time than HC during both the 0-back and 2-back load. | 0-back task: BD-I showed lower degree centrality(DC) in the mPFC, the precentral gyrus(PreCG),the ACC(BA32),the cuneus (CUN), the calcarine (CAL), the precuneus(PCUN),and the lingual gyrus (LING) compared with HC. 2-back task: BD-I had higher DC in the inferior parietal cortex (IPL) and lower DC in the mPFC, the PreCG, the PoCG, the DLPFC, the paracentral lobule (PCL), the MTG, the hippocampus (HIP),the CUN and the cerebellum than HC. | 9★ | 5 | High |
| Alonso-Lana et al., 2019 | BD-I:26/HC:26 | 39.19(12.33)/40.21 (10.97) | 42.3%/42.3% | All medicated. Mania (n=26): MS n=19, AP n=22, AD n=2; Euthymia (n=26): MS n=23, AP n=17, AD n=3.The impact of the drug was not explicitly reported in Results section.. | Mania，Euthymia | N-back | 1.BD-I (Mania) vs HC: There was significantly worse on both 1-back and 2-back. 2.BD-I (Euthymia) vs HC: There was no longer significantly different. | BD-I(Mania) vs HC(2-back): Reduced activation in the left DLPFC/precentral cortex (peak in BA6), and the superior parietal cortex extending medially to include the superior part of the precuneu (peak in BA17). Failure of deactivation in the vmPFC (peak in BA11). BD-I(Euthymia) vs HC(2-back): Failure of deactivation in the vmPFC (BA11). Longitudinally (Mania vs Euthymia,2-back): Activation increased in the left DLPFC/precentral cortex (BA6) and the superior parietal cortex extending to the superior part of the precuneus bilaterall(peak in BA7) from mania to euthymia, while failure of deactivation in the vmPFC persisted. | 8★ | 5 | High |
| Wu et al., 2014 | BD-I: 20 / HC: 29 | 27.85(6.39)/22.72(5.14) | 50.0%/48.3% | All medicated:AP n=11, AD n=7, Li n=10, anticonvulsants n=8, BZD n=1. (No significant correlation found between medication dosage and PCC-mPFC effective connectivity.) | Not specified (HAMD/YMRS reported) | N-back | BD-I had significantly lower accuracy on the 2-back task compared to HC. No significant difference in reaction time. | 2-back task: BD-I showed significantly hyperactivity in the left posterior cingulate cortex (PCC) and left mPFC compared to HC. Effective Connectivity: The effective connectivity from PCC to MPFC was positive in BD-I , but negative in HC. The effective connectivity from MPFC to PCC was negative in BD-I, positive in HC. | 8★ | 5 | High |
| Bertocci et al., 2012 | BD-I:18/HC:16 | 31.94 (8.54)/32.76 (6.50) | 100%/100% | 14 medicated: AD n=6, AP n=9, MS n=11, BZD n=3 (numbers reflect medication classes, not mutually exclusive); 4 unmedicated.The patient's neural activity did not show a significant effect of psychotropic drugs | Depressed | EFNBACK | No significant differences in accuracy or reaction time on the 2-back task. | 2-back (neutral faces vs no faces): BD-I showed significantly greater activation than HC in the bilateral putamen. (Within-group patterns: HC deactivated, BD-I activated these regions). 2-back (happy faces vs no faces): BD-I showed greater right putamen activity than HC. | 7★ | 4 | High |
| Mullin et al., 2012 | BD-I:22 / HC:19 | 31.68(8.96)/32.54(6.56) | 63.6% / 57.9% | All medicated: MS n=16, AP n=12, AD n=9, BZD n=3.The impact of the drug was not explicitly reported in Results section. | Euthymic | EFNBACK | No significant differences in accuracy or reaction time between BD-I and HC groups. | 2-back vs 0-back(no-distracter): BD-I showed reduced activation in right dlPFC(BA9), right dACC(BA31), bilateral inferior parietal cortex(BA40), and right putamen compared to HC. 2-back fearful-face vs neutral-face: BD-I showed increased activation in left dlPFC(BA9), bilateral inferior parietal cortex(BA40), right amygdala, and right putamen compared to HC. Effective connectivity: To the 2-back fearful-face distracter condition, BD-I showed significantly lower preceding EC from rostral/dACC to amygdala than HC. To the 2-back happy-face distracter condition, BD-I showed significantly greater preceding EC from rostral/dACC to amygdala than HC. | 8★ | 4 | High |
| McKenna et al., 2014 | BD-I:23 / HC:23 | 45.31(9.45)/44.78(10.59) | 65.2%/65.2% | All medicated: MS n=17, AP n=12, AD n=11, BZD n=10; 17 on polytherapy Medication load analysis showed that higher medication load was associated with increased BOLD signal in prefrontal regions (bilateral medial PFC, right IFG, left thalamus) during encoding. | Euthymic | DMTS | BD-I were less accurate than HC only for four-syllable pseudowords. | Encoding interval: BD-I exhibited attenuated activation compared to HC in the bilateral mPFC (BA10), right dlPFC/IFG/middle frontal gyrus (BA10/45/46), bilateral postcentral gyrus/premotor cortex (BA3/4/6), bilateral precuneus/cingulate gyrus/caudate/putamen/thalamus/insula (BA7/13/23), and left middle temporal gyrus/posterior cingulate gyrus (BA21/30). Maintenance interval: BD-I demonstrated hyperactivation relative to HC in the right postcentral gyrus (BA2), right middle occipital cortex/middle temporal cortex (BA19/37), and bilateral cuneus (BA18). Functional connectivity: The connection between the bilateral mPFC and right IFG in BD-I was stronger than HC. | 8★ | 4 | High |
| McKenna et al., 2015 | BD-I: 26 / HC: 36 | 45.21 (10.10)/46.25 (11.54) | 69% / 53% | All medicated: MS n=19 (Li n=8), AP n=14, AD n=13, BZD n=11; 19 on polytherapy. Medication analysis revealed no significant associations between medication load (or medication class) and any neuroimaging or behavioral measure | Euthymic | DMTS | Working Memory Accuracy: BD-I group was significantly lower than HC group. | Encoding Interval: BD-I patients showed significantly reduced activation in the left DLPFC (BA9/46) compared to HC. | 8★ | 4 | High |
| Stegmayer et al., 2015 | BD-I:17 / HC:17 | 38.2 (9.9) / 33.9 (11.5) | 44% / 61% | 15 medicated: 12 on MS (Li n=5, Val n=5, CBZ n=2, LTG n=2), 4 on AP, 6 on AD, 4 on BZD; 3 unmedicated. The impact of the drug was not explicitly reported in Results section. | Euthymic | DMTS | No significant differences in accuracy or reaction time between BD-I and HC groups. | PPI analysis:Compared with HC, BD-I showed a significantly reduced negative functional connectivity between the right amygdala and the right precentral gyrus, the right frontal eye field ,the right intraparietal cortex , and the (pre)-SMA. | 7★ | 5 | High |
| Huang et al., 2019 | BD-I:41/HC: 58 | 31.3(12.0)/29.2(9.5) | 41.5% /36.2% | All medicated：AP n=28, AD n=10, MS=34, Anxiolytic(includes BZD) n=10.The dose of antipsychotic drugs (CPZ equivalent dose) was not significantly associated with brain activation | Psychotic features(mood state not specified) | DMTS | Accuracy: No significant difference between groups. Reaction Time: BD-I was significantly slower than HC. | Encoding Interval: BD-I patients showed reduced activation compared to HC in the frontal eye fields (FEF), intraparietal sulcus (IPS), and superior parietal lobule (SPL). Late delay Maintenance：BD-I patients showed reduced activation in the FEF and IPS compared to HC. | 9★ | 5 | High |
