# Supplementary material for: Working memory network dysfunction in bipolar I vs. bipolar II disorder: a systematic review of task-fMRI evidence
Source: Front Psychiatry. 2026 Jun 16;17:1800042. doi: 10.3389/fpsyt.2026.1800042 (PMC13320638; doi:10.3389/fpsyt.2026.1800042)
Supplement: Supplementary file 3 [file Table3.docx]

**Supplementary Table 3 fMRI Quality Control Score**

| **Author and Year** | **Design** | **Correction Score (0/1)** | **Correction Evidence** | **Motion Control Score (0/1)** | **Motion Control Evidence** | **Sample Score (0/1)** | **Sample Evidence (final N)** | **Behavior Score (0/1)** | **Behavior Evidence** | **Task Engagement Score (0/1)** | **Task Engagement Evidence** | **Checklist Total (0–5)** | **Whole Brain Uncorrected Only (Yes/No)** |
| --- | --- | --- | --- | --- | --- | --- | --- | --- | --- | --- | --- | --- | --- |
| Alonso-Lana et al., 2016 | Cross-sectional | 1 | FLAME stage 1 (z>2.3) with p<0.05 corrected for multiple comparisons (FSL FEAT). | 1 | Excluded participants with max abs movement >3.0 mm or avg abs movement >0.3 mm. | 1 | Final fMRI N: BD-I=50;HC=28. Exclusions: 10 BD-I (technical acquisition/processing problems) + 1 BD-I (excessive motion). | 1 | In-scanner performance reported using d′ for 1-back and 2-back with group comparisons/statistics. | 1 | Within-group activations reported: 2-back vs baseline in controls shows bilateral DLPFC and other canonical WM regions. | 5 | No |
| Alonso-Lana et al., 2019 | Longitudinal | 1 | Cluster-level inference with family-wise corrected p=0.05 using Gaussian random field; cluster-forming threshold z=2.3. | 1 | Excluded max abs movement >3.0mm or avg abs movement >0.3mm; motion parameters included as nuisance covariates. | 1 | Final fMRI N: BD-I=26 (scanned twice: mania/euthymia); HC=26 (two sessions). Exclusions: negative d′ on 1-back/2-back excluded; excessive motion excluded (max >3.0 mm or mean >0.3 mm). | 1 | Reports in-scanner task performance (d′) for 1-back and 2-back with group comparisons and longitudinal change. | 1 | Within-group activations in healthy controls include bilateral DLPFC and superior parietal cortex during 2-back vs baseline. | 5 | No |
| Bertocci et al., 2012 | Cross-sectional | 1 | ROI-based correction: AlphaSim Monte Carlo simulations (1000) used to compute minimum cluster size thresholds per ROI to control Type I error at 0.05; voxelwise p≤0.05 (post-hoc voxelwise p≤0.016). | 1 | Motion parameters from realignment included as covariates of no interest in first-level models. | 1 | Final fMRI N: BD-I=18; HC=16. Incorrect-response trials excluded; motion parameters modeled as nuisance covariates. | 1 | In-scanner performance reported: mean accuracy 92.82% and reaction time; no significant group differences reported with statistics. | 1 | Primary within-task EFNBACK contrasts were reported (2-back: emotional faces [neutral/happy/fear] minus 2-back: no-face distracter), with activation results presented in this article. | 4 | No |
| Brooks et al., 2015 | Cross-sectional | 1 | Whole-brain cluster correction: height threshold Z>2.0 with cluster probability p<0.05 corrected for whole-brain multiple comparisons using Gaussian random field theory. | 1 | Excluded participants with excessive motion (>3 mm translation peak-to-peak over 117 functional images); additionally included 6 motion-correction parameters as covariates of no interest in GLM. | 1 | Final fMRI N: BD-II=19; HC=19. Exclusions: 4 BD-II + 3 HC (excessive movement >3 mm and/or magnetic susceptibility dropout) + 1 BD-II (poor behavioral performance) | 1 | Reports in-scanner n-back performance (accuracy + reaction time) with repeated-measures ANOVA and group comparisons. | 1 | Whole-brain within-group results for the parametric 0/1/2-back load contrast and ROI analyses showed robust task-related activation (incl. bilateral DLPFC) in both controls and BD-II | 5 | No |
| Caseras et al., 2015 | Cross-sectional | 1 | ROI correction: 3DClustSim (AFNI) with threshold p=0.001 used to determine minimum cluster size for corrected p<0.05 within each ROI (amygdala=9 vox; accumbens=13; DLPFC=26). | 1 | Excluded participants with excessive head motion (>3 mm displacement in any direction); motion correction applied using MCFLIRT; motion parameters did not differ between groups and were accounted for in GLM analyses. | 1 | Final fMRI N: BD-I=16; BD-II=19; HC=19. Eligibility/exclusion criteria reported; exclusions: 2 BD-I (uncertain euthymic status at scanning) + 1 HC (poor task understanding; excluded from behavioral/fMRI). | 1 | In-scanner behavioral performance reported (accuracy, RT) with ANOVA and group interactions; pairwise comparisons reported. | 1 | Reports expected WM network activation: 2-back+no-distracters > 0-back+no-distracters shows activity within working memory network; distracters also increase activity within WM network . | 5 | No |
| Dell'Osso et al., 2015 | Cross-sectional | 1 | FWE correction at p=0.05 within ROI (middle frontal gyrus; WFU PickAtlas); additionally p=0.05 FWE-corrected for whole-brain comparisons. | 1 | Realigned to first volume; excluded excessive motion (>3.5 mm translation or >3° rotation); motion parameters included as covariates. | 1 | Final fMRI N: BD-I=15; BD-II=13; HC=27 (2 BD-I excluded—excessive head motion) | 1 | Behavioral performance (accuracy, reaction time) reported with repeated-measures ANOVA; no diagnosis effect (accuracy F(4,92)=0.52, p=0.71; RT F(4,92)=0.58, p=0.67). | 1 | Main effect of WM load observed in bilateral middle frontal gyri with FWE-corrected. | 5 | No |
| Delvecchio et al., 2015 | Cross-sectional | 1 | Voxel-level FWE correction used for suprathreshold clusters (P < 0.05). | 0 | Foam padding/forehead strap used and images realigned; however no explicit motion exclusion thresholds and no statement that motion parameters were included as GLM regressors. | 1 | Final fMRI N: BD-I=41; HC=46. Eligibility/exclusion criteria reported; post-acquisition exclusions not specified | 1 | In-scanner performance evaluated as reaction time and accuracy; group differences tested (ANOVA with Bonferroni-corrected pairwise comparisons) and task performance reported. | 1 | Task contrast (3-back vs 0-back) yields expected activation in canonical WM circuitry, including lateral prefrontal cortex (inferior/middle frontal gyrus). | 4 | No |
| Dima et al., 2016a | Cross-sectional | 1 | Whole-brain inference with FWE correction at p<0.05; cluster size k>20. | 0 | Preprocessing reports realignment/normalization/smoothing but no explicit motion exclusion threshold or motion regressors stated. | 1 | Final fMRI N: BD-I=41; HC=46. Eligibility/exclusion criteria reported; post-acquisition exclusions not specified | 1 | Reports in-scanner 2-back accuracy and reaction time. | 1 | Main 2-back>baseline effect activates canonical WM network (lateral PFC and parietal cortex). | 4 | No |
| Dima et al., 2016b | Cross-sectional | 1 | Second-level random-effects analysis; suprathreshold clusters identified using family-wise error correction (P<0.05) with cluster extent k>20. | 1 | In SPM8 analyses, six movement parameters entered as nuisance covariates (reported for both face task and working-memory GLM). | 1 | Final fMRI N: BD-I=41; HC=46. Eligibility/exclusion criteria reported; post-acquisition exclusions not specified | 1 | Reports in-scanner task performance for working memory (3-back accuracy and response time) in Table 1 and analyzes group effects on accuracy/RT. | 1 | Working-memory contrast defined as 3-back > 0-back; imaging results involve canonical WM regions (e.g., inferior/middle frontal gyrus, ACC; DCM network includes DLPFC, ACC, parietal cortex). | 5 | No |
| Frangou et al., 2017 | Cross-sectional | 1 | Second-level suprathreshold clusters identified with voxel-wise FWE correction p<0.05. | 1 | Motion QC: excessive interscan motion >4mm translation or >4° rotation; 6 motion parameters entered as nuisance covariates; no group differences. | 1 | Final fMRI N: BD-I=30; HC=30. Eligibility/exclusion criteria reported; post-acquisition exclusions: none (motion QC performed; no subjects excluded). | 1 | Task performance reported (3-back % correct and response time) by group. | 1 | 3-back vs 0-back patterns involve lateral/frontopolar prefrontal cortex and dorsal parietal cortex—canonical WM network nodes. | 5 | No |
| Goikolea et al., 2019 | Cross-sectional | 1 | Whole-brain inference thresholded at P < .05 (FWE) for fMRI (and PPI). | 1 | Motion QC: no exclusions for excessive motion (>4 mm translation or >4° rotation); motion parameters entered as nuisance covariates. | 1 | Final fMRI N: BD-I=31; HC=31. Eligibility/exclusion criteria reported. Post-acquisition exclusions: negative d′ criterion applied (numbers not specified); no participants excluded for motion (>4 mm translation, >4° rotation). | 1 | In-scanner task performance quantified with d′; Table 1 reports 2-back performance with between-group comparison; negative d′ participants excluded. | 1 | Canonical WM network main effect: 2-back vs baseline shows fronto-parietal activations (e.g., middle frontal gyrus/dlPFC and inferior/superior parietal lobules). | 5 | No |
| Huang et al., 2019 | Cross-sectional | 1 | ROI: Bonferroni-adjusted α ≤ 0.01 (5 tests). Whole-brain: small-volume cluster correction; clusters surviving p < .05 FWE (cluster-level) with voxel-wise p < .001. | 1 | Preprocessing includes slice timing and motion correction; six motion parameters included as covariates in first-level GLM. | 1 | Final fMRI N: BD-I=41; HC=58. Eligibility/exclusion criteria reported. Post-acquisition exclusions reported: HC=1 and BD-I=1 ineligible/withdrew; HC=3 and BD-I=7 excluded for WM accuracy <60%. | 1 | Behavioral performance reported (d′ accuracy and RT). Accuracy did not differ between groups; RT showed group effects. | 1 | Whole-brain WM>Control activations show distributed frontoparietal/subcortical regions across groups; ROIs derived from canonical spatial WM regions (FEF, IPS, SPL, MFG, AI). | 5 | No |
| Jogia et al., 2012 | Cross-sectional | 1 | Second-level suprathreshold clusters identified using Family Wise Error (FWE) correction, P ≤ 0.05. | 1 | Checked excessive interscan motion (≤4 mm translation, ≤4° rotation); no subjects excluded and no significant group differences in motion. | 1 | Final fMRI N: BD-I=36; HC=37. Eligibility/exclusion criteria reported. Post-acquisition exclusions: none (motion QC threshold ≥4 mm translation/≥4° rotation; no subjects were excluded). | 1 | In-scanner n-back accuracy (% correct), errors, and response time reported; Table I reports accuracy/RT per load; no diagnosis effect on performance (P≥0.18). | 1 | Main effect of task activates canonical WM regions (middle/inferior frontal gyrus and parietal cortex/inferior parietal lobe) in both groups (Table III). | 5 | No |
| McKenna et al., 2014 | Cross-sectional | 1 | Whole-brain cluster-threshold correction using Monte Carlo simulations; reliable activation required 22 contiguous voxels (1408 μL) with voxelwise p≤0.01, yielding corrected p=0.01. | 0 | Motion artifact correction via co-registration is described, but no explicit motion exclusion threshold and no statement that motion parameters were included as GLM regressors. | 1 | Final fMRI N: BD-I=23; HC=23. Eligibility/exclusion criteria reported; post-acquisition exclusions not specified. | 1 | In-scanner behavioral results reported (accuracy and response latency) with group effects and load effects. | 1 | Encoding interval shows task-related activation in canonical WM regions in healthy participants (PFC and basal ganglia/thalamus; frontoparietal/subcortical pattern). | 4 | No |
| McKenna et al., 2015 | Cross-sectional | 1 | Whole-brain cluster-size correction (cluster threshold method): voxels thresholded at p≤0.01; clusters considered reliable if they met a Monte-Carlo–derived contiguous-voxel threshold, providing corrected p=0.01. | 0 | Preprocessing mentions motion co-registration/realignment but does not report motion exclusion criteria and does not state that motion parameters were entered as GLM regressors. | 1 | Final fMRI N: BD-I=26; HC=36. Eligibility/exclusion criteria reported; post-acquisition exclusions not specified. | 1 | Within MRI, participants completed two WM tasks; accuracy from both WM tasks was averaged to create a working-memory composite, and group comparisons were conducted (t-tests). | 1 | Task produced robust WM-related task effects used to define functional ROIs (bilateral DLPFC and supramarginal gyri) as the most task-responsive subregions across participants; delayed match-to-sample task previously validated/published. | 4 | No |
| Mullin et al., 2012 | Cross-sectional | 1 | AlphaSim/FWE correction reported (cluster threshold) within ROIs. | 0 | Realignment/unwarping performed; motion parameters included as nuisance regressors, but no reportable motion QC (thresholds/exclusions/scrubbing) described. | 1 | Final fMRI N: BD-I=22; HC=19. Eligibility/exclusion criteria reported; task-performance exclusion criterion specified (accuracy <70%; 0 BD-I and 1 HC excluded); motion-based post-acquisition exclusions not specified. | 1 | In-scanner performance reported; no significant group differences / main effects described. | 1 | Contrast targets WM network; canonical regions (dlPFC/dACC/parietal) reported in results for n-back contrasts. | 4 | No |
| Pomarol-Clotet et al., 2011 | Cross-sectional | 1 | Whole-brain cluster-level corrected (GRF), z=2.3; cluster P=0.05 corrected (FSL FEAT). | 1 | Excluded for motion: max abs movement >3.0 mm or mean abs movement >0.3 mm; 11/45 excluded for excessive movement. | 1 | Final fMRI N: BD-I=29; HC=46. Eligibility/exclusion criteria reported; post-acquisition exclusions: 11 BD-I (excessive motion) + 5 BD-I (poor compliance; negative d’). | 1 | In-scanner performance reported (d′): patients worse than controls on 1-back and 2-back (both P<0.0001); analyses also covaried d′. | 1 | Main 2-back vs baseline shows canonical WM activations including DLPFC and parietal regions in controls (and also in manic group). | 5 | No |
| Rodríguez-Cano et al., 2017 | Cross-sectional | 1 | Cluster-level inference with family-wise corrected P=.05 (Gaussian random field); z=2.3 cluster-forming threshold. | 1 | Motion QC with exclusion thresholds: max absolute movement >3.0 mm OR average absolute movement >0.3 mm excluded. | 1 | Final fMRI N: BD-I=26; HC=26. Eligibility/exclusion criteria reported; post-acquisition exclusion criteria specified (negative d′; motion thresholds), but number excluded not reported. | 1 | In-scanner task performance reported (d′) for 1-back and 2-back; group differences tested (ANOVA + Tukey HSD); patient groups not significantly different from each other. | 1 | Main task contrast (2-back vs baseline) shows canonical WM pattern in controls: bilateral DLPFC + parietal activations with mPFC/precuneus deactivations. | 5 | No |
| Stegmayer et al., 2015 | Cross-sectional | 1 | Within-group connectivity maps thresholded at p<0.05 FDR-corrected; between-group contrasts reported at p=0.001 uncorrected (two-sample t-tests, within a priori WM mask). | 1 | Participants exceeding 2.5 mm movement were excluded (2/38 removed); six motion parameters were included as nuisance regressors in first-level models. | 1 | Final fMRI N: BD-I=17; HC=17. Eligibility/exclusion criteria reported; post-acquisition exclusions: 1 BD-I + 1 HC (motion >2.5 mm). | 1 | In-scanner behavior reported: performance rates 86.7% (patients) vs 91.1% (controls), p<0.1; reaction times not significantly different (p=0.37). | 1 | Task described as an established verbal delayed match-to-sample paradigm that reliably activates a canonical verbal WM network (e.g., Broca’s area, premotor cortex, intraparietal cortex). | 5 | No |
| Verdolini et al., 2023 | Cross-sectional | 1 | Whole-brain cluster-level GRF correction: z=2.3, family-wise corrected p=0.05. | 1 | Excluded max absolute movement >3.0mm or average absolute movement >0.3mm; motion parameters included as nuisance covariates. | 1 | Final fMRI N: BD-I=31; HC=31. Eligibility/exclusion criteria reported; post-acquisition exclusions described (performance d′-based; motion threshold defined), but the exact number excluded after acquisition is not fully reported. | 1 | Reported task performance (d′) for 1-back and 2-back with group comparisons; excluded negative d′ values. | 1 | Within-group activation/deactivation maps were reported for the primary n-back contrasts (1-back > baseline, 2-back > baseline, and 2-back > 1-back), showing robust task-related fronto-parietal activations and default-mode deactivations (mPFC and PCC/precuneus), supporting WM task engagement. | 5 | No |
| Wu et al., 2014 | Cross-sectional | 1 | Whole-brain deactivation (2-back) reported with p<0.05 FWE-corrected, cluster>100; between-group comparisons used p<0.001 uncorrected. | 1 | Realignment with max translation/rotation <=1.0 mm (x,y,z). | 1 | Final fMRI N: BD-I=20; HC=29. Eligibility/exclusion criteria reported; post-acquisition exclusions not specified (motion constraint ≤1.0 mm translation/rotation). | 1 | Accuracy differs among groups (F=9.943, p<0.001); reaction time not different across groups (p>0.05). | 1 | The study modeled fixation, resting, 0-back, and 2-back conditions separately and computed 2-back > resting and resting > 2-back contrasts. Whole-brain analyses showed consistent task-related deactivation during 2-back, including the left PCC and mPFC (FWE-corrected), providing neural evidence of task engagement/load manipulation. | 5 | No |
| Xi et al., 2023 | Cross-sectional | 1 | Post hoc voxel-wise comparisons used P<0.05 FDR correction (ANOVA mask; ANOVA threshold P<0.005 cluster-level). | 1 | A rigorous head motion control process was implemented, including head motion correction, regression of head motion parameters, and interpolation of anomalous time points. It was confirmed that there were no significant differences in inter-frame displacement among the three groups (p > 0.05). | 1 | Final fMRI N: BD-I=31; HC=80. Eligibility/inclusion/exclusion criteria reported . Additional analysis-level exclusions noted: participants excluded if no voxels survived DC thresholding (r>0.25), with condition-specific analyzed N (rest: BD-I=25, HC=59; 0-back: BD-I=28, HC=79; 2-back: BD-I=26, HC=80). | 1 | In-scanner accuracy & RT reported and analyzed with group×task repeated-measures ANOVA; key group differences reported. | 1 | The WM paradigm comprised 0-back and 2-back load conditions. Whole-brain voxel-wise degree centrality (DC) maps were computed and significant DC differences were reported across groups under both 0-back and 2-back loads , indicating load-dependent task-related network engagement. | 5 | No |
